# Comparison of the abilities of universal, super, and specific DNA barcodes to discriminate among the original species of Fritillariae cirrhosae bulbus and its adulterants

**DOI:** 10.1371/journal.pone.0229181

**Published:** 2020-02-13

**Authors:** Qi Chen, Xiaobo Wu, Dequan Zhang

**Affiliations:** 1 College of Pharmacy and Chemistry, Dali University, Dali, China; 2 Institute of Materia Medica, Dali University, Dali, China; 3 Key Laboratory of Yunnan Provincial Higher Education Institutions for Development of Yunnan Daodi Medicinal Materials Resources, Dali, China; Kunming Institute of Botany, Chinese Academy of Sciences, CHINA

## Abstract

Fritillariae cirrhosae bulbus is a famous type of traditional Chinese medicine used for cough relief and eliminating phlegm. The medicine originates from dried bulbs of five species and one variety of *Fritillaria*. Recently, immature bulbs from other congeneric species, such as *F*. *ussuriensis*, have been sold as adulterants of Fritillariae cirrhosae bulbus in medicine markets owing to the high price and limited availability of the genuine medicine. However, it is difficult to accurately identify the bulbs from different original species of Fritillariae cirrhosae bulbus and its adulterants based on traditional methods, although such medicines have different prices and treatment efficacies. The present study adopted DNA barcoding to identify these different species and compared the discriminatory power of super, universal, and specific barcodes in *Fritillaria*. The results revealed that the super-barcode had strong discriminatory power (87.5%). Among universal barcodes, *matK* provided the best species resolution (87.5%), followed by ITS (62.5%), *rbcL* (62.5%), and *trnH*-*psbA* (25%). The combination of these four universal barcodes provided the highest discriminatory power (87.5%), which was equivalent to that of the super-barcode. Two plastid genes, *ycf1* and *psbM*-*psbD*, had much better discriminatory power (both 87.5%) than did other plastid barcodes, and were suggested as potential specific barcodes for identifying *Fritillaria* species. Phylogenetic analyses indicated that *F*. *cirrhosa* was not a “good” species that was composed of multiple lineages, which might have affected the evaluation of the discriminatory ability. This study revealed that the complete plastid genome, as super barcode, was an efficient and reliable tool for identifying the original species of Fritillariae cirrhosae bulbus and its adulterants.

## Introduction

*Fritillaria* L. is one of the most important genera in the family Liliaceae, and it includes approximately 140 species of perennial herbaceous plants worldwide [1,2]. Almost all known species in this genus grow in temperate regions of the Northern Hemisphere [3]. There are 24 species in China, most of which have medicinal value and are used for cough relief and eliminating phlegm, such as *F*. *cirrhosa* D. Don, *F*. *ussuriensis* Maxim., *F*. *walujewii* Regel, *F*. *thunbergii* Miq., and so on [4–6]. According to the Chinese Pharmacopoeia (2015), there are five traditional Chinese medicines that originate from the dried bulbs of *Fritillaria* species. Among them, Fritillariae cirrhosae bulbus (also known as “Chuanbeimu” in traditional Chinese medicine) has been regarded the best throughout Chinese history. The original species used to make this type of medicine include five species and one variety, namely *F*. *cirrhosa*, *F*. *przewalskii* Maxim., *F*. *unibracteata* Hsiao et K.C. Hsia, *F*. *delavayi* Franch., *F*. *taipaiensis* P.Y. Li, and *F*. *unibracteata* var. *wabuensis* (S.Y. Tang et S.C. Yue) Z.D. Liu., S. Wang et S.C. Chen [7]. Recently, the market price of Fritillariae cirrhosae bulbus has been increasing sharply due to its limited output from wild habitats, and thus immature bulbs of other *Fritillaria* species (e.g., *F*. *ussuriensis*, *F*. *thunbergii*, *F*. *pallidiflora* Schrenk, etc.) have been sold in medicinal markets imitating the original Fritillariae cirrhosae bulbus. Genuine Fritillariae cirrhosae bulbus originating from bulbs with different botanical origins are difficult to identify based on the morphological characteristics and traditional methods. The bulbs from each original species obviously differ in price and might also differ in their medicinal treatment efficacy, which could seriously affect their health benefits to consumers [8–11]. Thus, it is extremely important to be able to accurately discriminate between Fritillariae cirrhosae bulbus and its adulterants, as well as among the different original species used to make these medicines.

DNA barcoding is a technology used for species identification that employs a short and standardized DNA region [12–15]. It has been proven to be an effective tool for rapid and accurate species discrimination [16–19]. Based on a large number of studies, three plastid loci (*trnH*-*psbA*, *matK*, and *rbcL*) and one ribosomal DNA spacer region (the internal transcribed spacer or ITS), or their combinations, were proposed as universal barcodes for plants that could discriminate most plant species [20–22]. However, for taxonomically complicated groups, such as *Fritillaria*, the use of these universal barcodes and their combinations has achieved lower success rates in species discrimination, except *matK* (this barcode had low success rate of PCR and Sanger sequencing in *Fritillaria*), due to insufficient sequence variation [23, 24].

The complete plastid genome was suggested as a super-barcode for identification of plant species, especially for taxonomically difficult taxa such as the genera *Citrus*, *Oncidium*, and *Gossypium* [25–27]. This genome has been also used successfully to explore phylogenetic relationships among plant taxa [28–30]. Typically, the plastid genome in angiosperms is circular, ranges in size from 72 to 217 kb, and has a quadripartite structure composed of a large single copy region (LSC), small single copy region (SSC), and a pair of inverted repeats (IRs) [31–34]. In contrast with the nuclear and mitochondrial genomes, the plastid genome is largely conserved among taxa in terms of its gene content, organization, and structure. Moreover, plastid genomes are maternally inherited and therefore appropriate for use in genetic engineering due to lack of cross-recombination [35,36]. These characteristics of the plastid genome render it suitable for species discrimination within complex plant taxa [37,38]. The results of recent studies also supported the strong discriminatory power of this super-barcode when used in species identification [39,40]. For the genus *Fritillaria*, complete plastid genomes were previously employed to evaluate phylogenetic relationships among some species and produced results that were supported by high bootstrap values [41–43]. However, those previous studies generally sequenced only one individual per species, and thus they could not effectively compare the intra- and inter-specific genetic distances in *Fritillaria* species. This leads to the question of whether species of *Fritillaria*, especially the original plants used to make Fritillariae cirrhosae bulbus and its adulterants, could be correctly identified at the species level by analyses of the complete plastid genome.

In the present study, we used the complete plastid genome sequence as a super-barcode to: identify the botanical origins of Fritillariae cirrhosae bulbus and its adulterants; compare the discriminatory power of the super-barcode with that of universal DNA barcodes and their combinations; and scan the highly variable gene regions as potential specific barcodes for species identification of Fritillariae cirrhosae bulbus. The present study provided abundant information for further development of super-barcodes and broadened the horizon over which other rapid and accurate molecular techniques for species identification in *Fritillaria* can be explored.

## Materials and methods

### Material sampling

**Fig 1:**
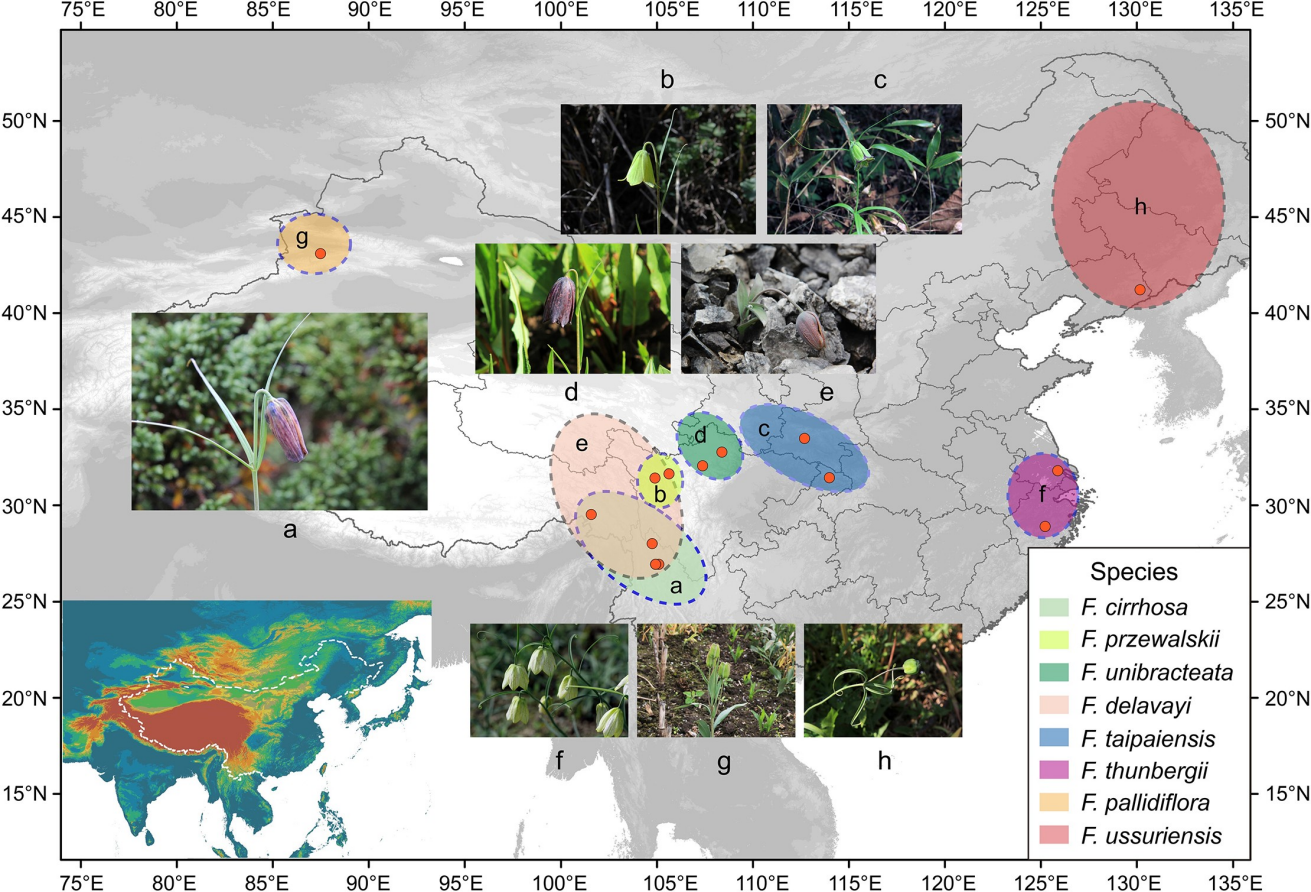
Distribution of the five original species of Fritillariae cirrhosae bulbus and three species of its adulterants. The distributional range of each species is drawn based on the records by Luo et al. [8,9] and herbarium specimens (http://www.cvh.ac.cn/). The a-f refer to these species and their distribution. Photos of the eight *Fritillaria* species studied are also added: **a**. *F*. *cirrhosa*; **b**. *F*. *przewalskii*; **c**. *F*. *taipaiensis*; **d**. *F*. *unibracteata*; **e**. *F*. *delavayi*; **f**. *F*. *thunbergii*; **g**. *F*. *pallidiflora*; **h**. *F*. *ussuriensis*.

A total of 32 individuals of *Fritillaria* representing the five original species of Fritillariae cirrhosae bulbus and three species of its adulterants, as well as an individual of *F*. *anhuiensis* S.C. Chen & S.F. Yin as an outgroup, were used in tree-building analysis in this study (Fig 1, S1 Table). Among these species, individuals of the five original species that produce the genuine medicine were collected from wild habitats in Southwest China, while those of the adulterants were collected from cultivated populations in provinces of Zhejiang, Jilin, and Xinjiang, China. None of the species from the genus *Fritillaria* are listed as the national protected plants in China (Information System of Chinese Rare and Endangered Plants, http://www.iplant.cn/rep/) and therefore their collection is allowed for scientific research. Fresh leaves were sampled from healthy, mature individuals in the field or cultivation bases and then dried by using silica gel. Meanwhile, 3–5 individuals with flowers were dug up and preserved as voucher specimens. Subsequently, geographic information (latitude, longitude, altitude, etc.) for the sampling locations was obtained by a Global Positioning System receiver (GPS; Garmin, Olathe, KS, USA). All voucher specimens of *Fritillaria* were identified and then deposited at the Herbarium of Medicinal Plants and Crude Drugs of the College of Pharmacy and Chemistry, Dali University, Dali, China (S1 Table).

#### DNA extraction, sequencing, and assembly

Total genomic DNA was extracted from about 100 mg of dried leaf material using a modified CTAB method [44,45]. The DNA content was checked by electrophoresis on 1.2% agarose gels, and its concentration was determined using a SmartSpec^TM^ Plus Spectrophotometer (Bio-Rad, Hercules, CA, USA). DNA extracts were then fragmented for the construction of 300 bp short-insert paired-end (PE) libraries and sequenced on an Illumina HiSeq 2000/2500 sequencer at the Beijing Genomics Institute (BGI, Shenzhen, China).

The raw data were filtered using Trimmomatic v.0.32 [46] with default settings. Paired-end reads in the clean data were then filtered and assembled into contigs using GetOrganelle.py [47] with *Fritillaria cirrhosa* (accession number: KF769143) as reference [48], calling the bowtie2 v., Blastn v., and SPAdes v.3.10 [49]. The *de novo* assembly graphs were visualized and edited using Bandage Linux dynamic v.8.0 [50], and then a whole or nearly whole circular plastid genome (plastome) was generated.

### Annotation and sequence submission

The plastomes were annotated by aligning them to the complete plastid genome sequence available in NCBI using MAFFT [48,51] with default parameters, which was coupled with manual adjustment using Geneious v.11.1.4 [52]. Circular genome visualization was generated with OGDRAW v.1.2 [53]. Furthermore, the ITS sequences were sequenced using Illumina sequencing technology and extracted from the raw data. The annotated plastid genomes of the nine *Fritillaria* species and their ITS sequences were submitted to the NCBI under the accession numbers MH588404-MH588436 for ITS sequences and those listed in Table 1 for the complete plastid genomes.

**Table 1:** Summary of complete plastid genomes obtained for the five original species of Fritillariae cirrhosae bulbus and three species of its adulterants, as well as the outgroup (*F*. *anhuiensis*).

Species	Code	Total length (bp)	Large single copy (LSC, bp)	Small single copy (SSC, bp)	Inverted repeat (IR, bp)	GC%	Number of genes	Accession number
*F*. *cirrhosa*	BM 1–1	151,546	81,402	17,542	26,301	37.0%	115	MH593342
	BM 1–2	151,546	81,402	17,542	26,301	37.0%	115	MH593343
	BM 2–1	151,998	81,755	17,545	26,349	37.0%	115	MH244906
	BM 2–2	151,605	81,467	17,534	26,302	37.0%	115	MH593344
	BM 3–1	152,035	81,794	17,541	26,350	36.9%	115	MH593345
	BM 3–2	152,035	81,794	17,541	26,350	36.9%	115	MH593346
*F*. *przewalskii*	BM 6–1	151,983	81,744	17,539	26,350	36.9%	115	MH244908
	BM 6–2	152,054	81,816	17,538	26,350	36.9%	115	MH593347
	BM 7–1	151,955	81,715	17,540	26,350	37.0%	115	MH593348
	BM 7–2	151,960	81,722	17,538	26,350	37.0%	115	MH593349
*F*. *unibracteata*	BM 8–1	151,058	81,339	17,539	26,090	37.0%	115	MH244909
	BM 8–2	151,057	81,338	17,539	26,090	37.0%	115	MH593350
	BM 9–1	151,012	81,295	17,537	26,090	37.0%	115	MH593351
	BM 9–2	151,078	81,398	17,538	26,071	37.0%	115	MH593352
*F*. *delavayi*	BM 10–1	151,853	81,602	17,513	26,369	37.0%	115	MH593353
	BM 10–2	151,854	81,603	17,513	26,369	37.0%	115	MH593354
	BM 10–3	151,854	81,603	17,513	26,369	37.0%	115	MH593355
*F*. *taipaiensis*	BM 11–1	151,707	81,451	17,552	26,352	37.0%	115	MH244910
	BM 11–2	151,518	81,268	17,546	26,352	37.0%	115	MH593356
	BM 12–1	151,741	81,478	17,561	26,351	37.0%	115	MH593357
	BM 12–2	151,741	81,478	17,561	26,351	37.0%	115	MH593358
	BM 12–3	151,741	81,478	17,561	26,351	37.0%	115	MH593359
*F*. *thunbergii*	BM 16–1	152,160	81,895	17,565	26,350	37.0%	115	MH244914
	BM 16–2	152,160	81,895	17,565	26,350	37.0%	115	MH593360
	BM 17–1	152,160	81,895	17,565	26,350	37.0%	115	MH593361
	BM 17–2	152,160	81,895	17,565	26,350	37.0%	115	MH593362
*F*. *pallidiflora*	BM 23–1	152,073	81,779	17,514	26,390	37.0%	115	MH593364
	BM 23–2	152,067	81,763	17,528	26,388	37.0%	115	MH593365
	BM 23–3	152,073	81,780	17,513	26,390	37.0%	115	MH593366
*F*. *ussuriensis*	BM 26–1	151,571	81,773	17,126	26,336	36.9%	115	MH593367
	BM 26–2	151,523	81,741	17,122	26,330	37.0%	115	MH593368
	BM 26–3	151,552	81,764	17,124	26,332	37.0%	115	MH593369
*F*. *anhuiensis*	BM 20–2	152,119	81,817	17,560	26,371	37.0%	115	MH593363

### Variable site analysis

After using MAFFT v.7.129 to align the plastid genome sequences, BioEdit software was used to adjust the alignment manually [51,54]. A sliding window analysis was conducted to determine the nucleotide variability (Pi) in the whole plastid genome using DnaSP v.6.11 [55]. The step size was set to 200 bp, with a 600 bp window length. Moreover, the DnaSP software was used to identify and quantify the insertions/deletions (indels), mutations, and nucleotide variability (Pi) in all aligned datasets. The p-distances, GC content, variable sites, and parsimony informative sites in the genomes were identified and analyzed by the software MEGA v.7.0.26 [56].

### Species discrimination of universal, super, and specific barcodes

To evaluate the success rates of species discrimination with each barcode, we used a tree-building method to analyze 14 datasets for each of the single regions examined and their combinations. All single regions, including universal DNA barcodes and highly variable loci, were extracted from the complete plastid genome sequences, except the ITS/ITS2 region. These datasets were aligned with MAFFT [51] and used to build neighbor-joining trees (NJ) based on p-distances in the software MEGA [56]. The plastome of *F*. *anhuiensis* (accession number: MH593363) was used as the outgroup in these tree-building analyses. Species were regarded as being successfully discriminated if all the individuals of a given species formed a monophyletic group [57].

## Results

### Plastid genome organization of *Fritillaria*

The 32 complete plastid genomes, consisting of a circular double-stranded DNA, ranged from 151,518 bp in *F*. *taipaiensis* (accession number: MH593356) to 152,073 bp in *F*. *pallidiflora* (accession number: MH593366). The genomes possessed a typical quadripartite structure, which comprised a pair of IRs (26,090–26,390 bp), LSC (81,295–82,085 bp), and SSC (17,122–17,565 bp) regions (Table 1, S1 Fig). Overall GC content of the complete plastid genomes was 36.9%-37.0% (Table 1). Moreover, a total of 115 genes were found, namely 78 protein coding genes, 30 tRNA genes, and 4 rRNA genes, as well as 3 pseudogenes (Table 2, S1 Fig). The protein coding genes present in the plastid genome of *Fritillaria* included 9 genes for large ribosomal proteins, 12 genes for small ribosomal proteins, 5 genes for photosystem I, 15 genes for photosystem II, and 6 genes for ATP synthase (Table 2, S1 Fig).

**Table 2:** Genes included in *Fritillaria* plastid genomes.

Category for gene	Group of genes	Name of genes
Self-replication	Large subunit of ribosome	*rpl2*^a^*, *rpl14*, rpl16*, *rpl20*, *rpl22*, *rpl23*^a^, *rpl32*, *rpl33*, *rpl36*
	Small subunit of ribosome	*rps2*, *rps3*, *rps4*, *rps7*^a^, *rps8*, *rps11*, *rps12*^a^*, *rps14*, *rps15*, rps16*, *rps18*, *rps19*
	DNA dependent RNA polymerase	*rpoA*, *rpoB*, rpoC1*, *rpoC2*
	rRNA gene	*rrn4*.*5*^a^, *rrn5*^a^, *rrn16*^a^, *rrn23*^a^
	tRNA gene	trnK-UUU*, *trnI-GAU*^I^*, *trnA-UGC*^a^*, trnG-GCC*, trnV-UAC*, trnL-UAA*, *trnS-UGA*, *trnS-GCU*, *trnS-GGA*, *trnY-GUA*, *trnC-GCA*, *trnL-CAA*^a^, *trnL-UAG*, *trnH-GUG*^a^, *trnD-GUC*, *trnfM-CAU*, *trnW-CCA*, *trnP-UGG*, *trnI-CAU*^a^, *trnR-ACG*^a^, *trnI-CAU*^a^, *trnE-UUC*, *trnT-UGU*, *trnF-GAA*, *trnQ-UUG*, *trnR-UCU*, *trnT-GGU*, *trnM-CAU*, *trnV-GAC*^a^, *trnN-GUU*^a^, *trnN-GUU*^a^, *trnV-GAC*^a^, *trnG-UCC*
Gene for photosynthesis	Subunits of photosystem Ⅰ	*psaA*, *psaB*, *psaC*, *psaI*, *psaJ*
	Subunits of photosystem Ⅱ	*psbA*, *psbB*, *psbC*, *psbD*, *psbE*, *psbF*, *psbH*, *psbI*, *psbJ*, *psbK*, *psbL*, *psbM*, *psbN*, *psbT*, *psbZ*
	Subunits of NADH-dehydrogenase	ndhA*, *ndhB*^I^*, *ndhC*, *ndhD*, *ndhE*, *ndhF*, *ndhG*, *ndhH*, *ndhI*, *ndhJ*, *ndhK*
	Subunits of cytochrome b/f complex	*petA*, petB*, petD*, *petG*, *petL*, *petN*
	Subunit for ATP synthase	*atpA*, *atpB*, *atpE*, atpF*, *atpH*, *atpI*
	Large subunit of rubisco	*rbcL*
Other genes	Translational initiation factor	*infA*
	Maturase	*matK*
	Protease	clpP*
	Envelope membrane protein	*cemA*
	Subunit of Acetyl-carboxylase	*accD*
	C-type cytochrome synthesis gene	*ccsA*
	Open reading frames (ORF,ycf)	*ycf1*, *ycf2*^a^, ycf3*, *ycf4*, *ycf15*^a^, *ycf68*^a^

The lowercase letter a in superscript after gene names indicates genes located in IR regions. Asterisks indicate intron-containing genes.

### Highly variable regions in plastid genome

**Fig 2:**
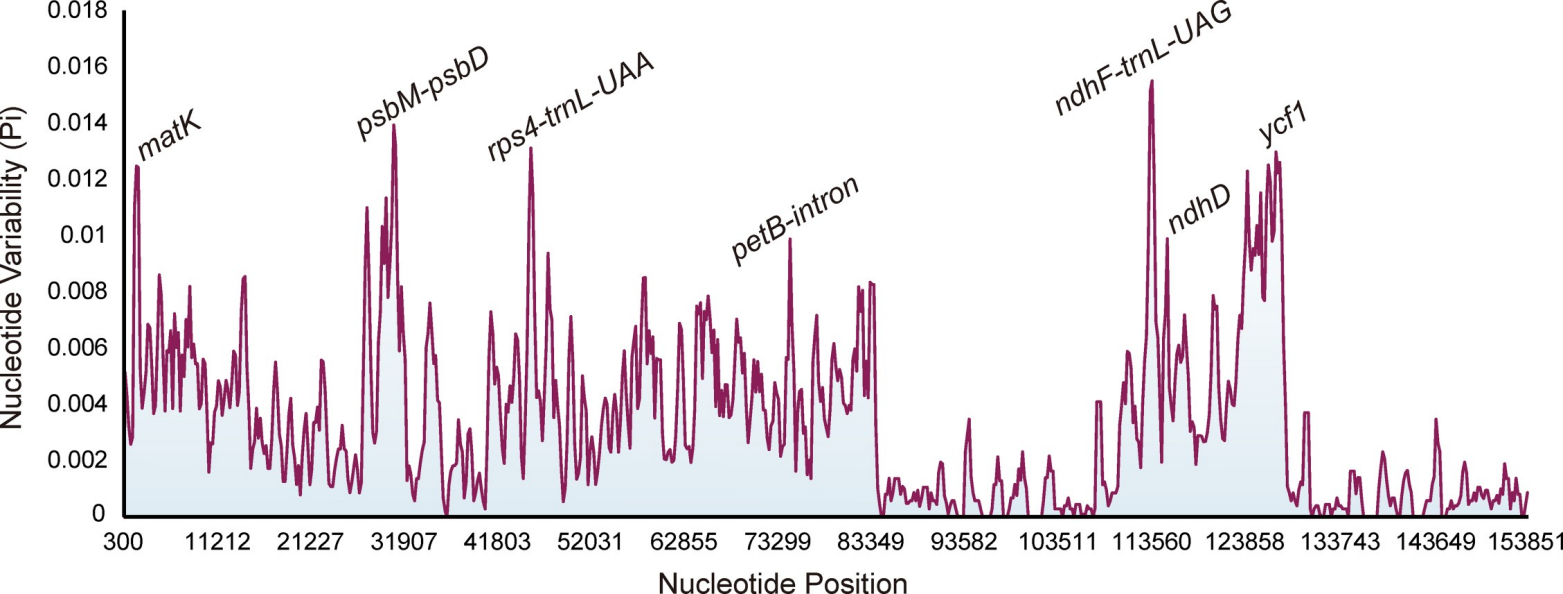
Sliding window analysis of 32 *Fritillaria* plastid genomes (window length: 600 bp, step size: 200 bp). X-axis: position of the midpoint of a window; Y-axis: nucleotide diversity of each window.

Seven highly variable regions from the plastid genomes, namely three intergenic regions (*psbM-psdD*, *rps4-trnL-UAA*, and *ndhF-trnL-UAG*), three gene regions (*matK*, *ndhD*, and *ycf1*), and one intron region (*petB-intron*), were selected as potential specific barcodes for use in species identification in *Fritillaria* (Fig 2). Among these regions, *ycf1* was the longest, followed by *psbM*-*psbD*, while *petB-intron* region was the shortest (Table 3). Furthermore, all highly divergent fragments were found in the LSC and SSC regions, whereas none were present in the IR regions.

**Table 3:** Analysis of the variability in different fragments and combination of fragments.

	No. sites	No. variable sites	No. parsimony information sites	No. mutations	No. InDels	Intraspecific distance		Interspecific distance		Nucleotide diversity (Pi)
						Range	Mean (± SE)	Range	Mean (± SE)	
Genome	154,404	2,667	2,436	2,449	4,273	0–0.0021	0.0005±0.0001	0.0007–0.0078	0.0037±0.0001	0.00337
HKLI	3,890	180	132	170	44	0–0.0052	0.0012±0.0002	0.0008–0.0191	0.0082±0.0006	0.00784
HKL	3,255	76	74	71	19	0–0.0034	0.0006±0.0002	0.0003–0.0117	0.0051±0.0001	0.00466
ITS2	239	52	22	55	9	0–0.0807	0.0094±0.0027	0–0.1211	0.0326±0.0013	0.03304
ITS	635	104	58	99	25	0–0.0338	0.0044±0.0011	0–0.0709	0.0235±0.0008	0.02560
*trnH-psbA*	253	17	17	16	19	0–0.0043	0.0010±0.0003	0–0.0598	0.0147±0.0009	0.01288
*matK*	1539	37	37	37	0	0–0.0039	0.0007±0.0002	0.0006–0.0123	0.0059±0.0001	0.00537
*rbcL*	1464	21	19	21	0	0–0.0027	0.0005±0.0001	0–0.0075	0.0033±0.0001	0.00295
*ndhF-trnL-UAG*	1,381	60	57	52	77	0–0.0052	0.0008±0.0002	0–0.0321	0.0112±0.0011	0.00994
*psbM-psdD*	3608	163	150	130	582	0–0.0058	0.0014±0.0003	0.0020–0.0202	0.0101±0.0002	0.00907
*rps4-trnL-UAA*	1,323	56	53	49	138	0–0.0061	0.0016±0.0003	0–0.0238	0.0098±0.0003	0.00892
*ycf1*	5,565	214	203	223	63	0–0.0044	0.0010±0.0002	0.0016–0.0240	0.0092±0.0003	0.00836
*petB-intron*	855	30	29	28	17	0–0.0050	0.0012±0.0002	0–0.0200	0.0078±0.0002	0.00703
*ndhD*	1,509	39	36	41	6	0–0.0020	0.0006±0.0001	0–0.0160	0.0056±0.0002	0.00502

HKL and HKLI represent a combination of three fragments of the plastid genome and a combination of four fragments of universal barcodes, respectively (H: *trnH-psbA*; K: *matK*; L: *rbcL*; I: ITS)

### Variability of the barcodes and their combinations

Among the regions and their combinations examined, the complete plastid genome clearly had the highest number of variable sites, parsimony-informative sites, and mutation sites (Table 3). Among the universal DNA barcodes, the ITS2 region was the most mutation-rich (Pi: 0.03304), followed by the ITS (0.02560), *trnH-psbA* (0.01288), and *matK* regions (0.00537), whereas the *rbcL* region (0.00295) was highly conserved (Table 3). Furthermore, among the highly variable regions, *ndhF-trnL-UAG* showed the highest variability (0.00994), and it was followed by *psbM-psdD* (0.00907), *rps4-trnL-UAA* (0.00892), *ycf1* (0.00836), *petB-intron* (0.00703), and *ndhD* (0.00502).

### DNA barcoding gap assessment

**Fig 3:**
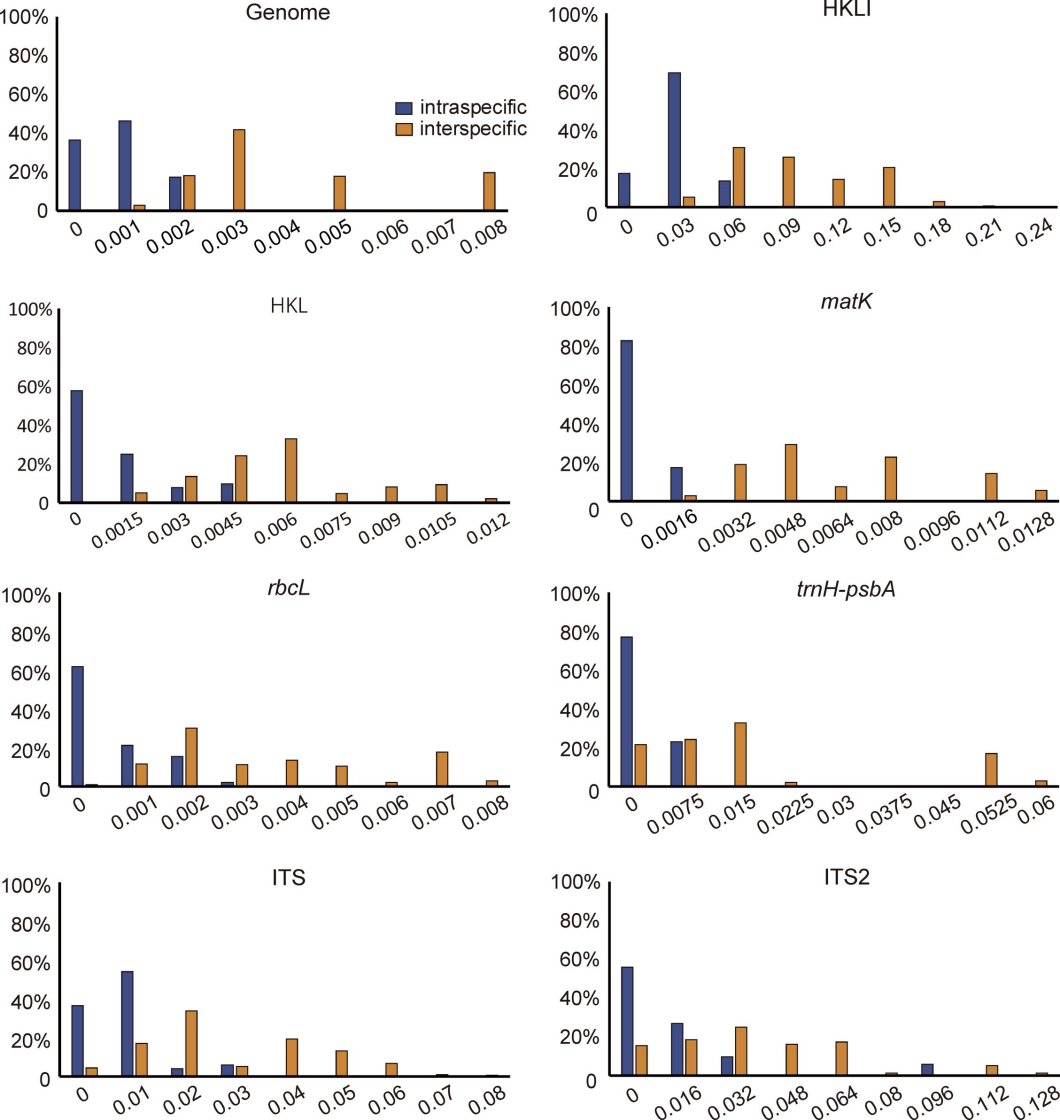
Histograms of the frequencies (y-axes) of pairwise inter- and intraspecific divergences calculated based on the p-distances (x-axes) of each single regions and their combinations. (H: *trnH-psbA*; K: *matK*; L: *rbcL*; I: ITS).

The inter- and intraspecific distances were calculated for each of the 14 datasets (Table 3). In these datasets, the ITS2 region exhibited the highest inter- and intraspecific distances (0.0326 and 0.0094, respectively), followed by the ITS (0.0235 and 0.0044), *trnH*-*psbA* (0.0147 and 0.0010) and *ndhF-trnL-UAG* (0.0112 and 0.0008), whereas these distances were the lowest for the *rbcL* (0.0033 and 0.0005). Meanwhile, the inter- and intraspecific distances of the complete plastid genome showed relatively low values (0.0037 and 0.0005, respectively) compared with those calculated for other datasets. Furthermore, the barcoding gap between inter- and intraspecific distances based on the p-distance model revealed that *matK* had the highest interspecific gap (divergence), but overlap between inter- and intraspecific distances existed for almost all single regions and their combinations, except for *matK* (Fig 3, S2 Fig).

### Discriminatory powers of all regions and their combinations

**Fig 4:**
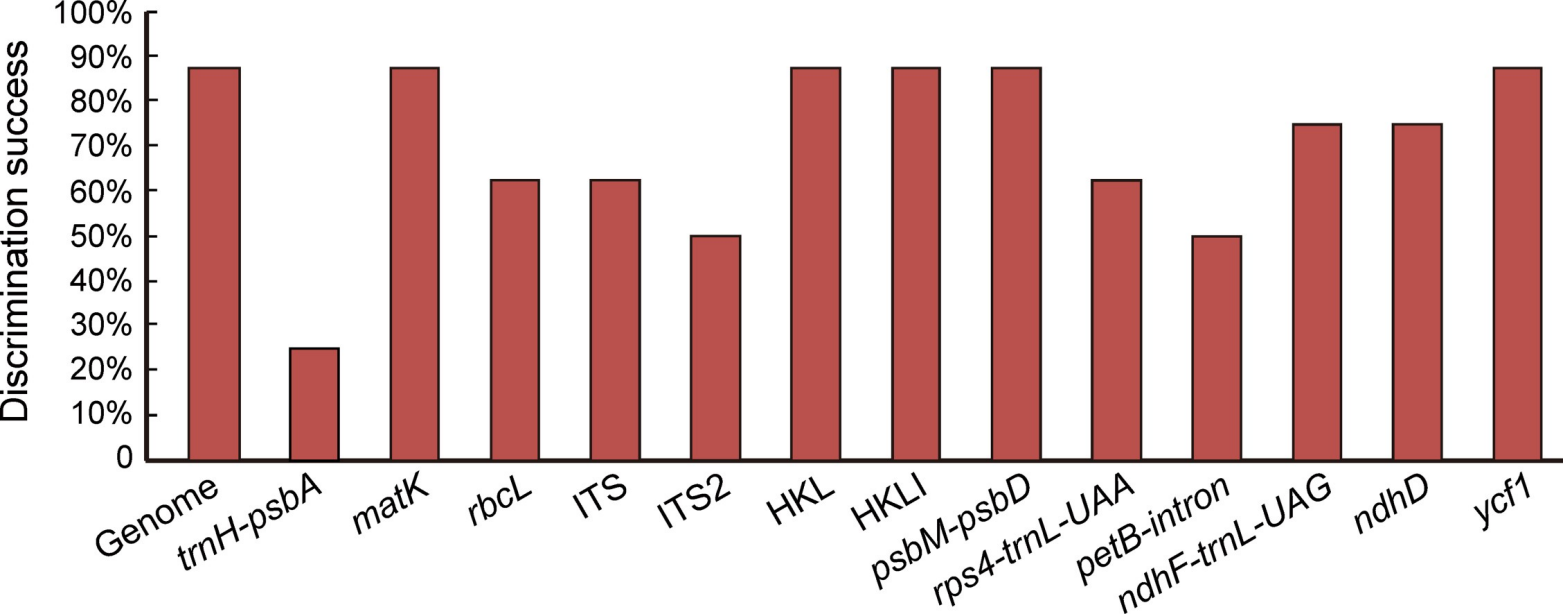
Species discrimination rate of all single fragments and their combinations based on the tree-building method. (H: *trnH-psbA*; K: *matK*; L: *rbcL*; I: ITS).

**Fig 5:**
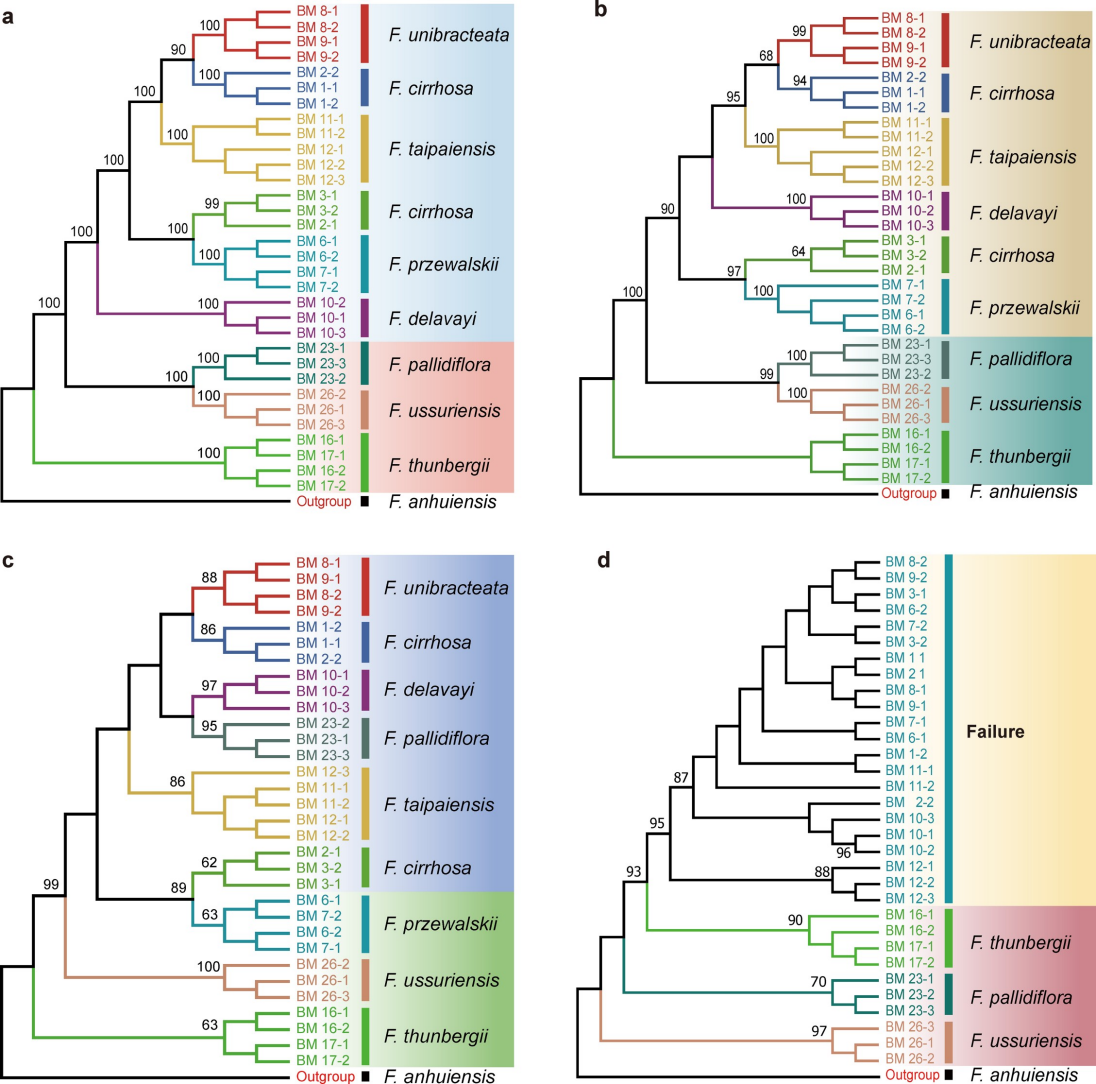
Neighbor-joining (NJ) tree of 33 specimens of *Fritillaria*, comprising 32 individuals of Fritillariae cirrhosae bulbus and its adulterants based on p-distances calculated for each barcode region: **a.** whole plastid genome; **b**. *ycf1*; **c.** *matK*; **d.** ITS2.

We calculated the species discrimination ability of each region and their combinations based on 14 datasets using tree-building methods (Fig 4). The super-barcode, comprised of complete plastid genomes, showed the highest power for species identification (87.5%), with strongest bootstrap values (Fig 5), except for *F*. *cirrhosa*, which is probably due to its polyphyletic nature. Among the universal DNA barcodes tested here, *matK* had the highest discriminatory power (87.5%), same as that of the super-barcode (Fig 4), followed by *rbcL* and ITS (both 62.5%). In contrast, *trnH*-*psbA* and ITS2, with relatively short DNA sequences (253 bp and 239 bp, respectively) and heavy overlaps between inter- and intraspecific distances, had relatively low success rates in distinguishing *Fritillaria* species (25% and 37.5%, respectively). Moreover, the combinations of the four barcodes (HKLI) or of the three plastid DNA regions (HKL) also showed the highest power for species discrimination (both 87.5%). Among the highly variable loci, *psbM*-*psbD* and *ycf1* had the highest species discrimination rates (both 87.5%), followed by *rps4*-*trnL-UAA* (75%), *ndhF*-*trnL-UAG ndhD*, and *petB*-*intron* (both 50%) (Fig 4).

According to the NJ trees, each of the DNA regions *psbM*-*psbD*, *ycf1*, and *matK* alone could be used to efficiently identify the original plants of Fritillariae cirrhosae bulbus and its adulterants. In contrast, the ITS, *ndhD*, *ndhF*-*trnL-UAG*, and *rps4*-*trnL-UAA* regions identified only the adulterant species from the genuine ones in the medicine. Moreover, the ITS2 could discriminate the adulterant from the genuine medicine, but could not effectively identify any of the original species of Fritillariae cirrhosae bulbus. Finally, the *trnH*-*psbA*, *rbcL*, and *petB-intron* regions could neither discriminate the adulterant medicine from the genuine nor their original species.

### Definition of *Fritillaria cirrhosa*

In the NJ trees inferred from the whole plastid genomes, other DNA regions, or combinations, the six individuals of *F*. *cirrhosa* collected from three locations were clustered into two distinct clades: one was placed close to *F*. *unibracteata* and the other was close to *F*. *przewalskii* with strong support values (Fig 5).

## Discussion

DNA barcoding has been demonstrated to be an efficient tool for identifying traditional medicines as well as their original species [23,58–60]. In the present study, this tool was used to discriminate among the five original species of Fritillariae cirrhosae bulbus and three of its adulterants.

### Performance of universal DNA barcodes

In plants, the term universal DNA barcodes, which are identified based on Sanger sequencing, generally refers to *rbcL* and *matK*, to be supplemented with *trnH-psbA* and ITS/ITS2 [21,22]. Among the four universal DNA barcodes, *matK* had the highest species discriminatory power and successfully distinguished all the original species of Fritillariae cirrhosae bulbus, except *F*. *cirrhosa*. In contrast, ITS2 and *trnH-psbA* showed the lowest species resolution and failed to correctly identify the original species of Fritillariae cirrhosae bulbus, although these two barcodes were previously suggested as core barcode for species identification in traditional Chinese medicines [22,61]. ITS/ITS2 correctly discriminated the genuine medicine from the adulterants (Fig 5, S3 Fig). This result was nearly consistent with that of our previous study [24]. In fact, ITS/ITS2 were rich in variable sites and nucleotide diversity (Pi) compared with the studied plastid DNA regions (Table 3), but the obvious overlap between inter- and intraspecific distances in such regions among and within *Fritillaria* species might impact their discriminating ability (Fig 3).

It should be noted that the *matK* sequences used for analysis were obtained from the complete plastid genome, not by Sanger sequencing. If the obstacles to Sanger sequencing of *matK* are resolved, *matK* will become the ideal candidate barcode for identifying the original species of Fritillariae cirrhosae bulbus, despite its lower nucleotide diversity (Pi) when compared with that of other regions. The best performance of this barcode might be attributed to the existence of a clear barcoding gap (Table 3, Fig 3). The *matK* gene has been confirmed to perform poorly in discriminating species of *Fritillaria* and other genera such as *Primula* [62] and *Garcinia* [63] because of its low success rates in PCR amplification and Sanger sequencing [24]. However, next-generation sequencing (NGS) technology could resolve the aforementioned deficiency of the Sanger method, as it was revealed in the present study that this marker has satisfactory discriminatory power. Therefore, we propose that NGS technology should be adopted for DNA sequencing of *matK* to overcome the disadvantages of the Sanger sequencing method for such fragments.

Furthermore, combined barcodes generally perform better in species discrimination than single barcodes [23]. In the present study, combinations of the four barcodes (HKLI) or three plastid regions (HKL) also showed strong species discrimination abilities (87.5%) (Fig 5, S3 Fig). In short, multi-locus combination could raise discrimination ability for *Fritillaria* species and improve the reliability.

### Super-barcode–a crucial candidate DNA barcode in *Fritillaria*

Because of the low power for species discrimination of universal DNA barcodes, new methods are necessary to discriminate closely related species [23,37]. Complete plastid genomes are extremely rich in genetic variations and have been shown to be powerful tools for resolving the phylogenetic relationships of complex groups [30,42,43,64,65]. Their use can greatly improve the resolution at lower taxonomic levels in plant phylogeny, phylogeography, and population genetics; therefore, they were also proposed as a type of super DNA barcode that is likely to resolve the defects of the universal DNA barcodes [23,66]. In this study, plastid genomes of *Fritillaria* species, with lengths from 151,518 to 152,073 bp, provided abundant informative sites for species identification. As a result, this super-barcode identified almost all of the original species of Fritillariae cirrhosae bulbus and its adulterants with high bootstrap values, except for *F*. *cirrhosa* because of its possible polyphyletic nature (Fig 5). Herein, the complete plastid sequences in the present study were obtained from dried leaf materials using NGS methods. Nevertheless, the use of this super-barcode might face extreme challenges if one needed to extract DNA from specific materials, such as kiln-dried specimens or medicines. However, recent procedures have been developed that can use total DNA as a template for genome skimming to assemble plastid genome, which not only solves the problem of extracting plastid DNA from dried or even degraded materials but also simplifies the whole process [23,67,68]. As sequencing technology and bioinformatics continue to improve rapidly, super DNA barcode will become more popular and may eventually replace Sanger-based DNA barcoding. Thus, super DNA barcodes could be adopted as useful complements to universal DNA barcodes, especially in identifying closely related species.

### Specific DNA barcode–a trade-off between universal and super-barcode

The present study revealed that, when adopting the NGS method, the universal DNA barcodes were limited in their ability to discriminate species, except *matK* region, which showed high success rate for identifying species in *Fritillaria* (87.5%). Although the super-barcode exhibited high discriminatory power and sufficient reliability in this study, its use might be limited due to complications in data analyses and expensive sequencing costs. Therefore, it is of great importance to search for specific barcodes from highly variable regions that can be used as a trade-off between universal and super DNA barcodes.

Highly variable regions in the plastome could help to effectively resolve phylogenetic relationships and identify species within complicated groups [30,69]. In the present study, six highly variable regions were extracted from the complete plastid genomes. Among these regions, *ycf1* had the same high species discriminating ability (87.5%) as the combination of four markers (HKLI); similarly, *psdM*-*psbD* showed the strikingly high discriminatory power (87.5%) (Fig 4, Fig 5). In fact, *ycf1* had been successfully used to reconstruct the phylogenies of the family Orchidaceae [70] and the genus *Astragalus* [71], and it showed an extremely strong power to resolve interspecific relationships.

Therefore, we conclude that *ycf1* and *psdM*-*psbD* can be used as potential specific barcodes for *Fritillaria*. However, the overly long DNA sequences of these regions result in some difficulties during PCR amplification and DNA Sanger sequencing [72]. Therefore, designing suitable primers to amplify shorter sections of those regions (about 1000 bp) with more variation might be another approach to be used in Sanger sequencing.

### Delimitation of *F*. *cirrhosa* and its effect on species discrimination abilities of DNA barcodes

In the current study, the NJ trees generated using plastid genomes, universal barcodes, specific barcodes, and their combinations failed to resolve the samples of *F*. *cirrhosa* from different locations into one clade (Fig 5, S3 Fig). In the tree based on genome sequences, six individuals were divided into two different clades, one (BM1-1, BM1-2, and BM 2–2) was sister to *F*. *unibracteata*, but the other (BM2-1, BM 3–1, and BM 3–2) was placed close to *F*. *przewalskii*. These results indicated that *F*. *cirrhosa* is not a “good” species but contains multiple different lineages, which was also supported by the previous analysis of population genetics data based on amplified fragment length polymorphism (AFLP) markers (unpublished data). It is well known that *F*. *cirrhosa* has extremely complex variations in its morphology, especially its floral characteristics. According to the field survey, individuals from Lijiang (ZDQ15019) possess yellow-green tepals, but they are dark purple in individuals from Shangri-La (ZDQ13053). The complex morphology of this species unavoidably causes confusion in its taxonomy. Delimitation, as well as phylogeny, of *F*. *cirrhosa* and its closely related species is still controversial [8] and requires further study based on more samples and better markers. Thus, if the polyphyletic condition of this species is not considered, we could conclude that all of the examined fragments, including the whole plastid genomes, *matK*, *ycf1*, and *psbM-psbD*, as well as the combination HKLI, were able to discriminate all the original species of Fritillariae cirrhosae bulbus and its adulterants with a success rate of 100%.

## Conclusion

In the present study, 32 individuals from eight species, representing five species of the original plants of Fritillariae cirrhosae bulbus and three of its adulterants, were employed to compare the species discriminatory powers of universal, super, and specific DNA barcodes. The results revealed that the whole plastid genome used as a super-barcode exhibited a powerful ability to identify *Fritillaria* species, with high reliability. Among the universal barcodes, only *matK* could discriminate almost all the original species when NGS methods are employed. It should be noted that ITS2 separated genuine Fritillariae cirrhosae bulbus from its adulterants, but it could not correctly identify the original species. Among the highly variable regions examined, *ycf1* and *psbM-psbD* are considered the primary potential specific barcodes for *Fritillaria* species, but their successful sequencing using the Sanger method will depend on developing primers that will amplify the barcodes in sections. Moreover, NJ analysis based on complete plastid genomes, as well as other regions, revealed that *F*. *cirrhosa* was polyphyletic and the variations in its morphology requires further research at the population level. As the costs of NGS continue to decrease and data analysis methods are simplified, the use of super-barcodes might become the primary method for species discrimination in plants. Overall, the results in this study help to recognize species discrimination ability of super, universal, and specific barcodes in complex groups, and provide new knowledge to accurately identify the original plants of Fritillariae cirrhosae bulbus and its adulterants.

## Supporting information

S1 Fig Gene map of *Fritillaria* plastid genomes. Genes shown on the outside of the circle are transcribed clockwise, and genes shown on the inside of the circle are transcribed counter-clockwise. Genes belonging to different functional groups are color-coded. The darker gray color in the inner circle corresponds to the GC content, and the lighter gray color corresponds to the AT content. (PDF)
(PDF)

S2 Fig Histograms of the frequencies (y-axes) of pairwise intra- and interspecific divergences based on the p-distances (x-axes) calculated for single regions and their combinations that were not listed in Fig 3. (H: *trnH-psbA*; K: *matK*; L: *rbcL*; I: ITS) (PDF)
(PDF)

S3 Fig Neighbor-joining (NJ) tree generated for 33 individuals, including 32 from the original species of Fritillariae cirrhosae bulbus and its adulterants, based on analyses of single regions and their combinations that were not listed in the Fig 5. (H: *trnH-psbA*; K: *matK*; L: *rbcL*; I: ITS) (PDF)
(PDF)

S1 Table Collection information for the eight original species and one outgroup in *Fritillaria* in this study. (DOCX)
