# Supplementary material for: Comparison of the abilities of universal, super, and specific DNA barcodes to discriminate among the original species of Fritillariae cirrhosae bulbus and its adulterants
Source: PLoS One. 2020 Feb 13;15(2):e0229181. doi: 10.1371/journal.pone.0229181 (PMC7018091; doi:10.1371/journal.pone.0229181)
Supplement: S1 Fig — Genes shown on the outside of the circle are transcribed clockwise, and genes shown on the inside of the circle are transcribed counter-clockwise. Genes belonging to different functional groups are color-coded. The darker gray color in the inner circle corresponds to the GC content, and the lighter gray color corresponds to the AT content. (PDF) (PDF) [file pone.0229181.s001.pdf]

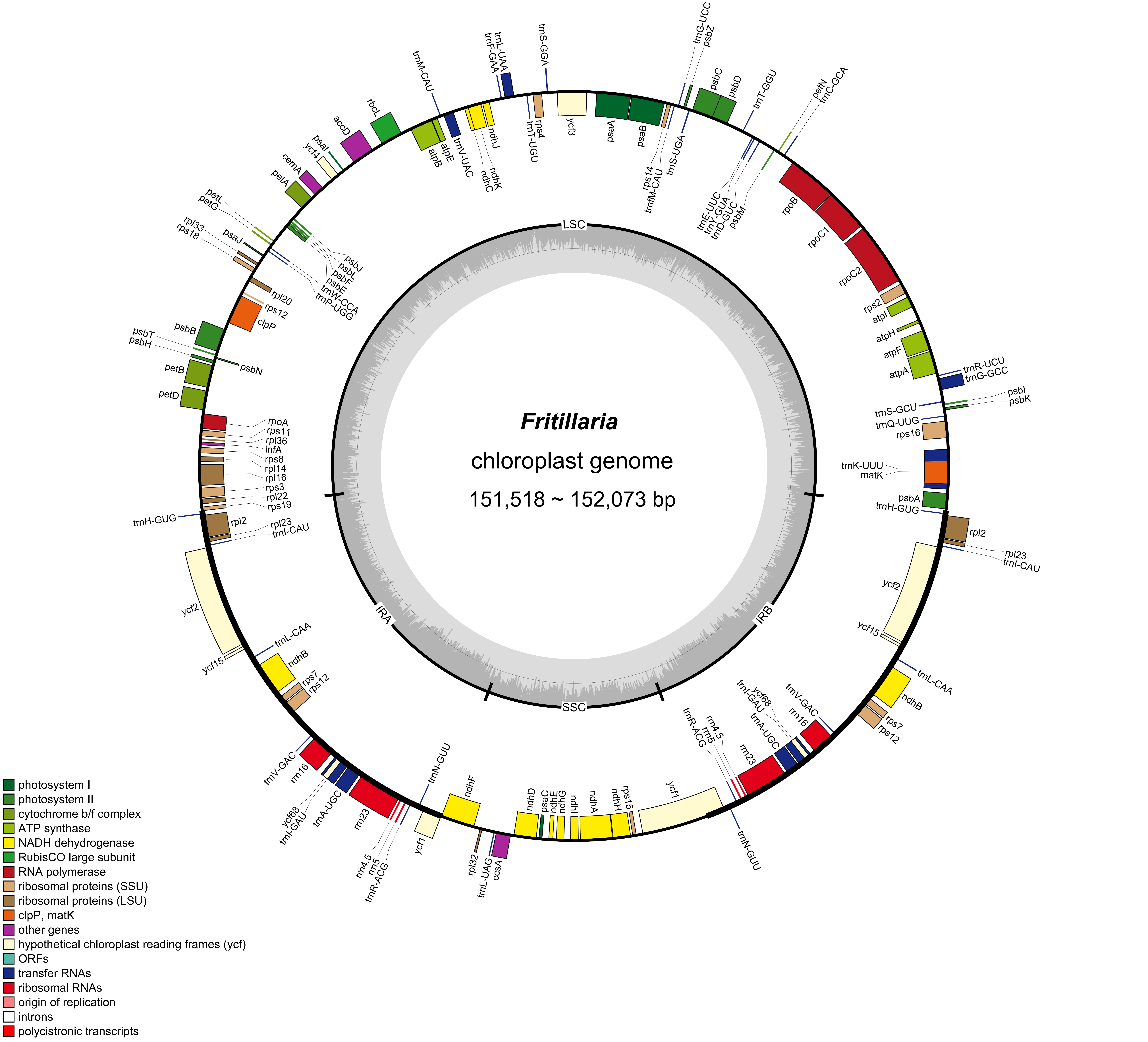
Fritillaria
chloroplast genome
151,518 ~ 152,073 bp
LSC
IRA
IRB
SSC
trnG-UCC
psbZ
psbC
psbD
trnT-GGU
petN
trnC-GCA
trnS-GGA
trnL-UAA
trnF-GAA
trnM-CAU
rbcL
accD
psaI
ycf4
cemA
petA
petL
petG
rpl33
rps18
psaJ
psbJ
psbL
psbF
psbE
trnW-CCA
trnP-UGG
rpl20
rps12
clpP
psbB
psbT
psbH
psbN
petB
petD
rpoA
rps11
rpl36
infA
rps8
rpl14
rpl16
rps3
rpl22
rps19
trnH-GUG
rpl2
rpl23
trnI-CAU
ycf2
ycf15
trnL-CAA
ndhB
rps7
rps12
trnV-GAC
rrn16
ycf68
trnI-GAU
trnA-UGC
rrn23
rrn4.5
rrn5
trnR-ACG
trnN-GUU
ycf1
ndhF
rpl32
trnL-UAG
ccsA
ndhD
psaC
ndhE
ndhG
ndhI
ndhA
ndhH
rps15
atpB
atpE
trnV-UAC
ndhC
ndhK
ndhJ
trnT-UGU
rps4
ycf3
psaA
psaB
rps14
trnfM-CAU
trnS-UGA
trnE-UUC
trnY-GUA
trnD-GUC
psbM
rpoB
rpoC1
rpoC2
rps2
atpI
atpH
atpF
atpA
trnR-UCU
trnG-GCC
psbI
psbK
trnS-GCU
trnQ-UUG
rps16
trnK-UUU
matK
psbA
photosystem I
photosystem II
cytochrome b/f complex
ATP synthase
NADH dehydrogenase
RubisCO large subunit
RNA polymerase
ribosomal proteins (SSU)
ribosomal proteins (LSU)
clpP, matK
other genes
hypothetical chloroplast reading frames (ycf)
ORFs
transfer RNAs
ribosomal RNAs
origin of replication
introns
polycistronic transcripts
