# Supplementary figures and images for: Comparison of the abilities of universal, super, and specific DNA barcodes to discriminate among the original species of Fritillariae cirrhosae bulbus and its adulterants
Source: PLoS One. 2020 Feb 13;15(2):e0229181. doi: 10.1371/journal.pone.0229181 (PMC7018091; doi:10.1371/journal.pone.0229181)

*ndhD*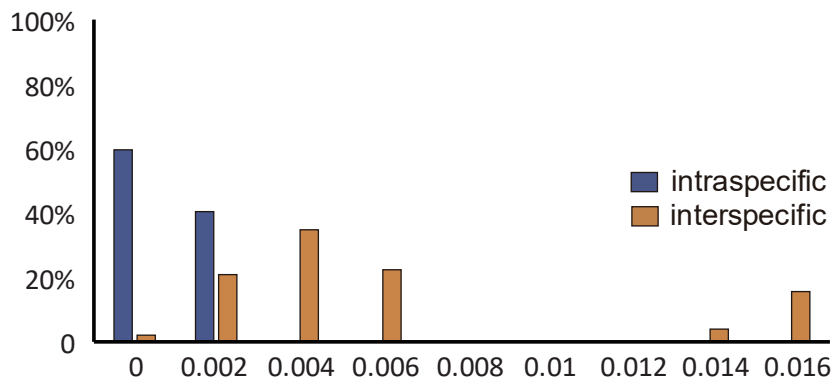*ycf1*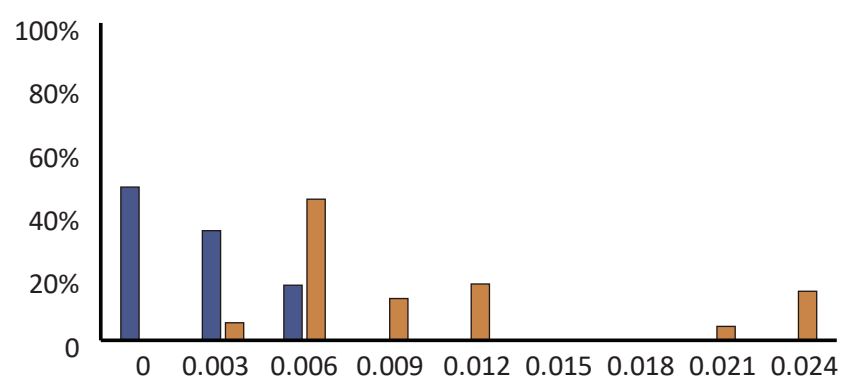*rps4-trnL-UAA*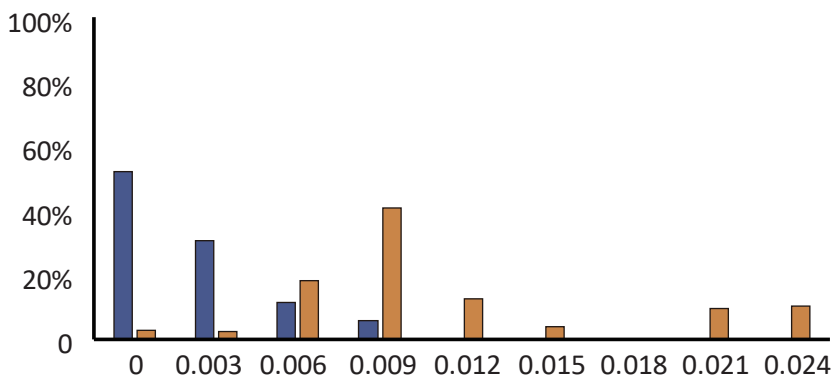*psbM-psdD*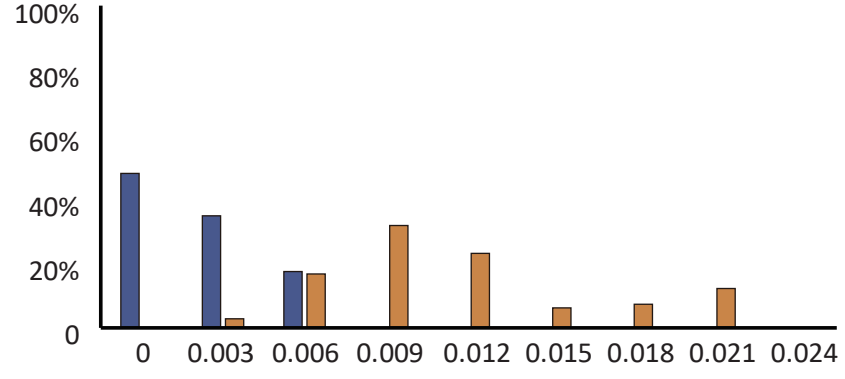*ndhF-trnL-UAG*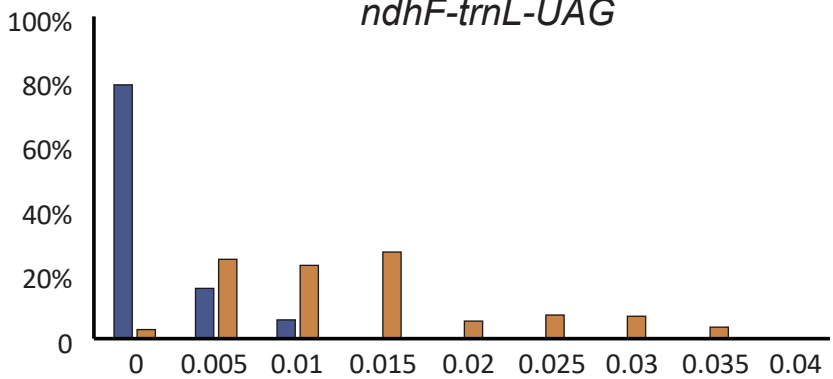*petB-intron*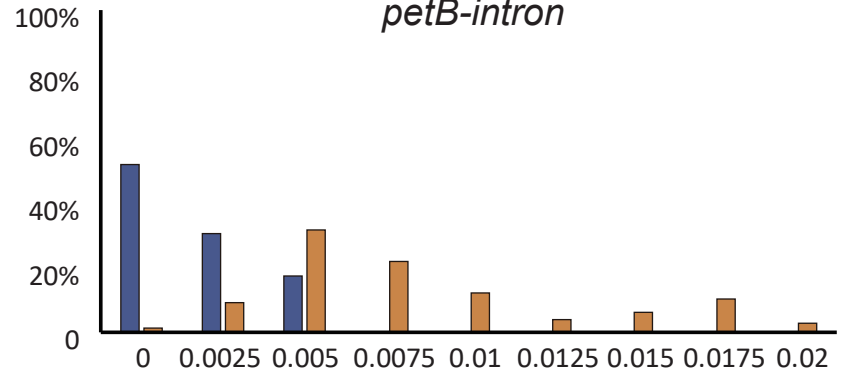

Supplement: S2 Fig — (H: trnH-psbA; K: matK; L: rbcL; I: ITS) (PDF) (PDF) [file pone.0229181.s002.pdf]

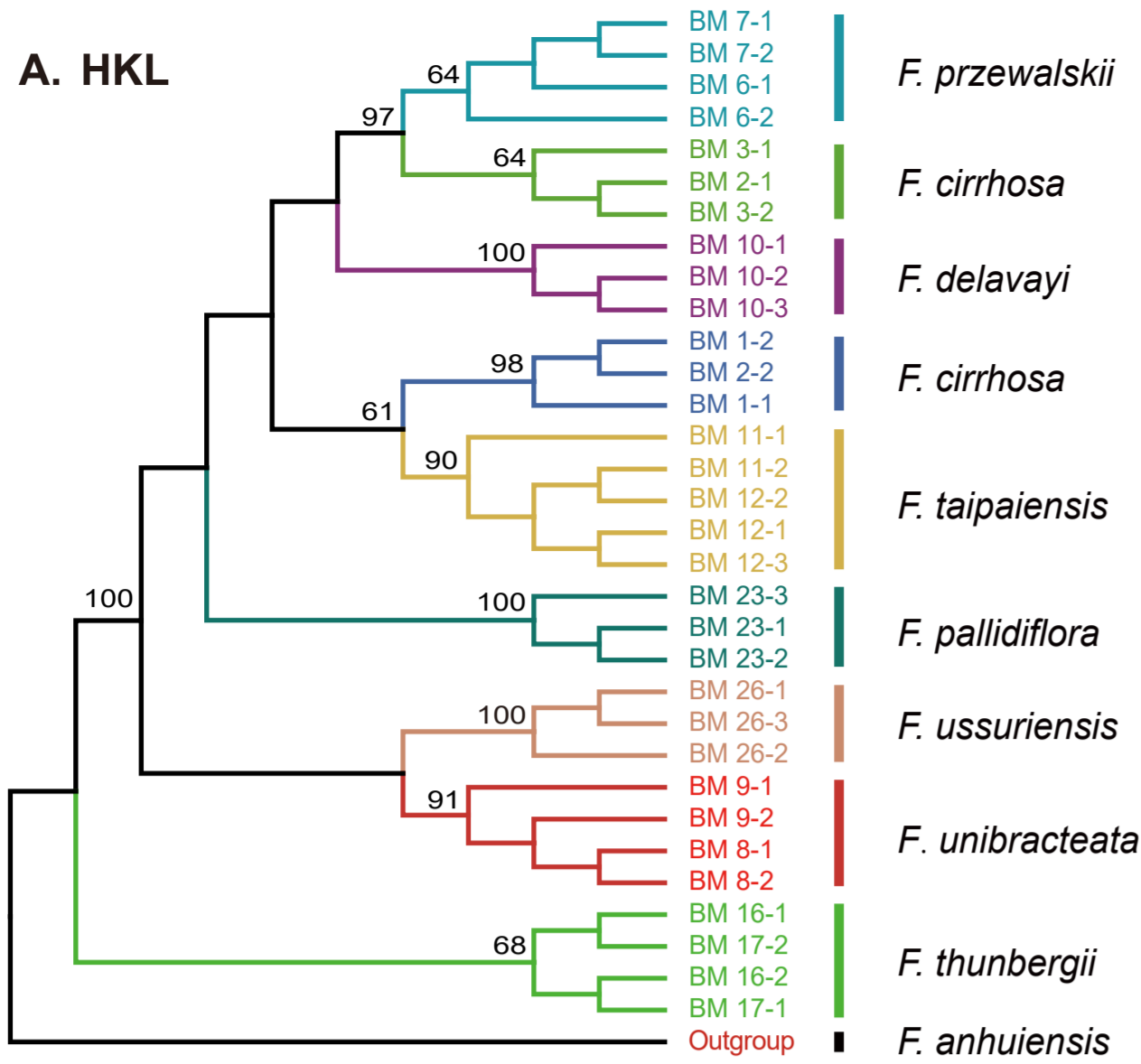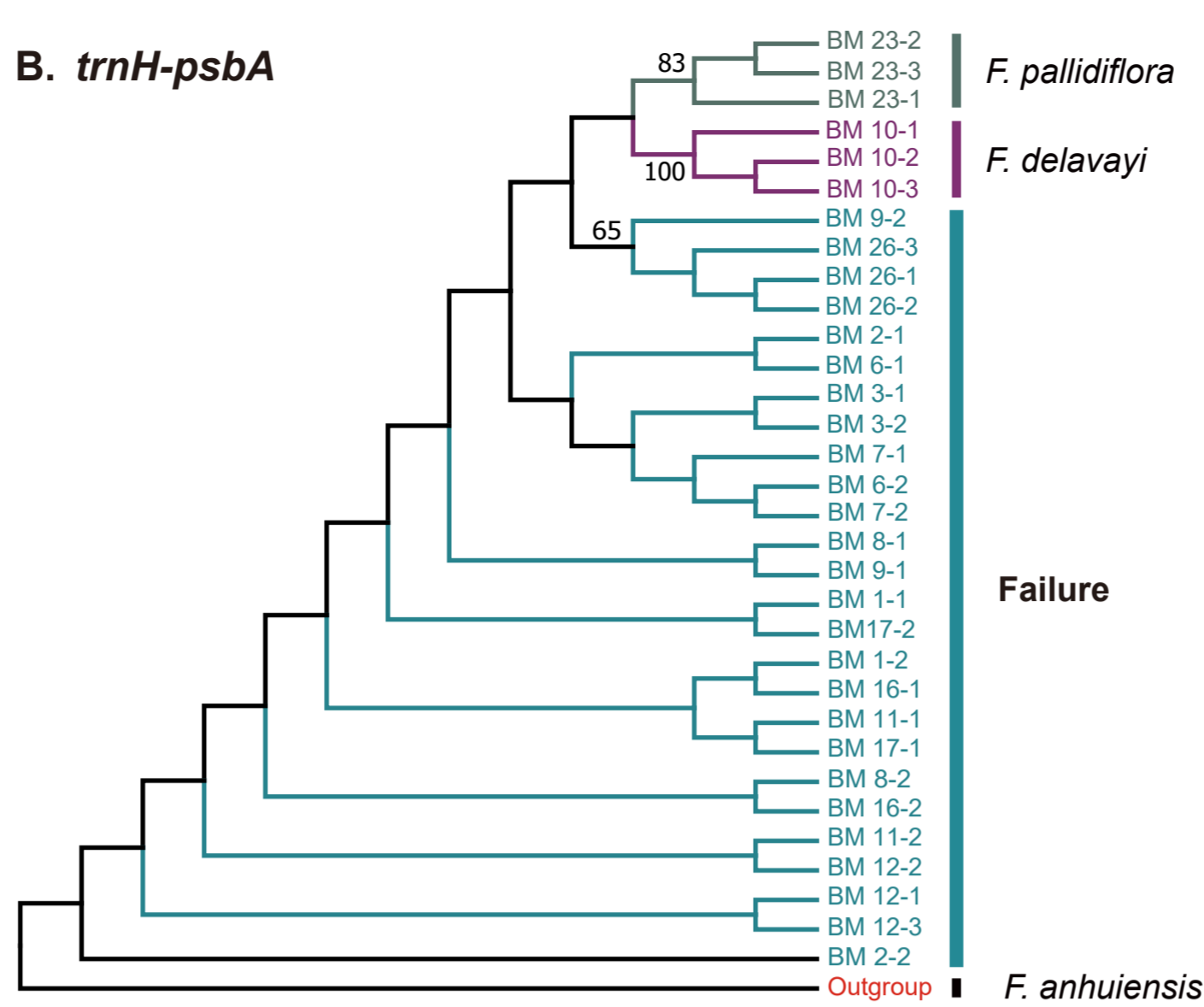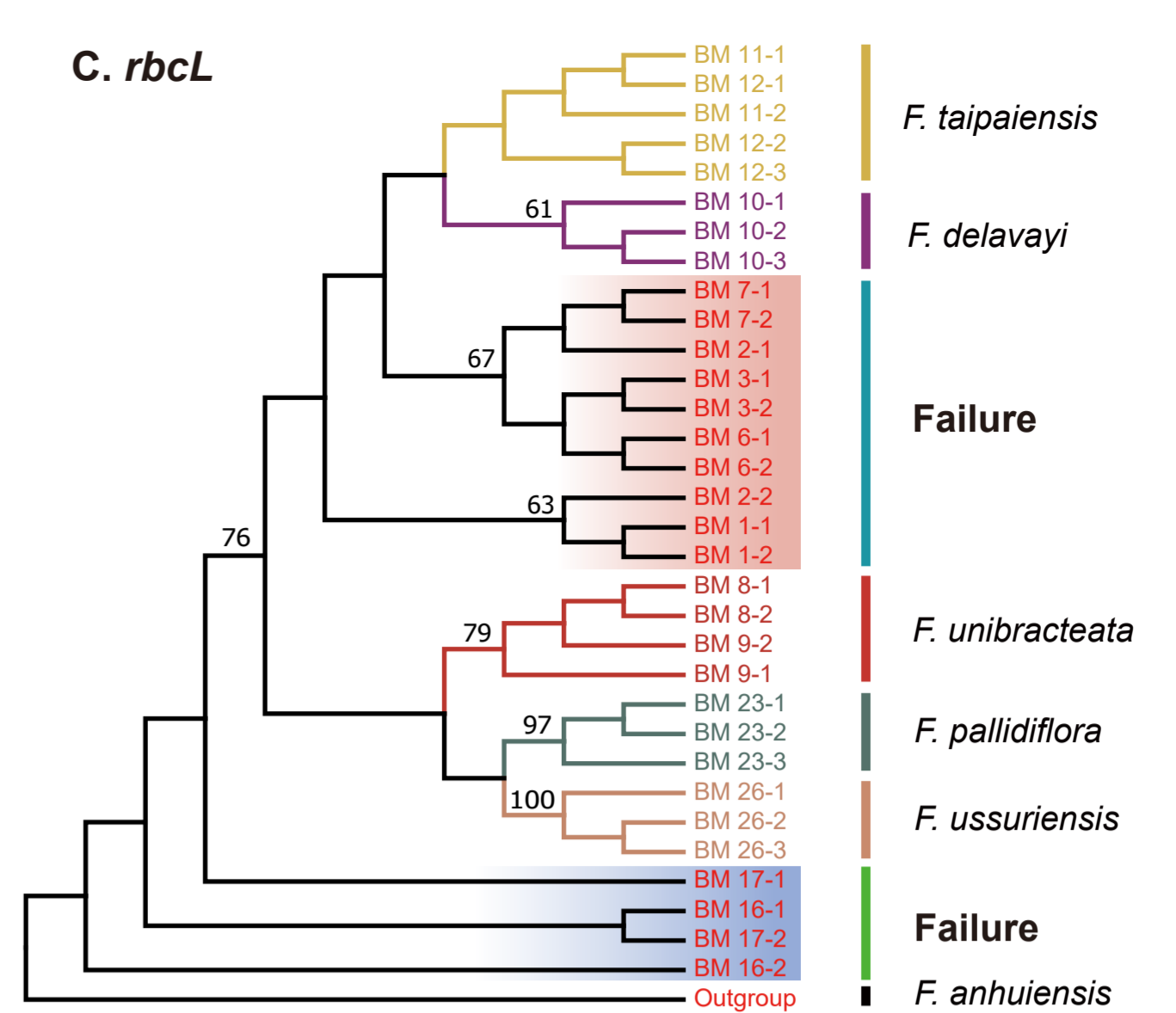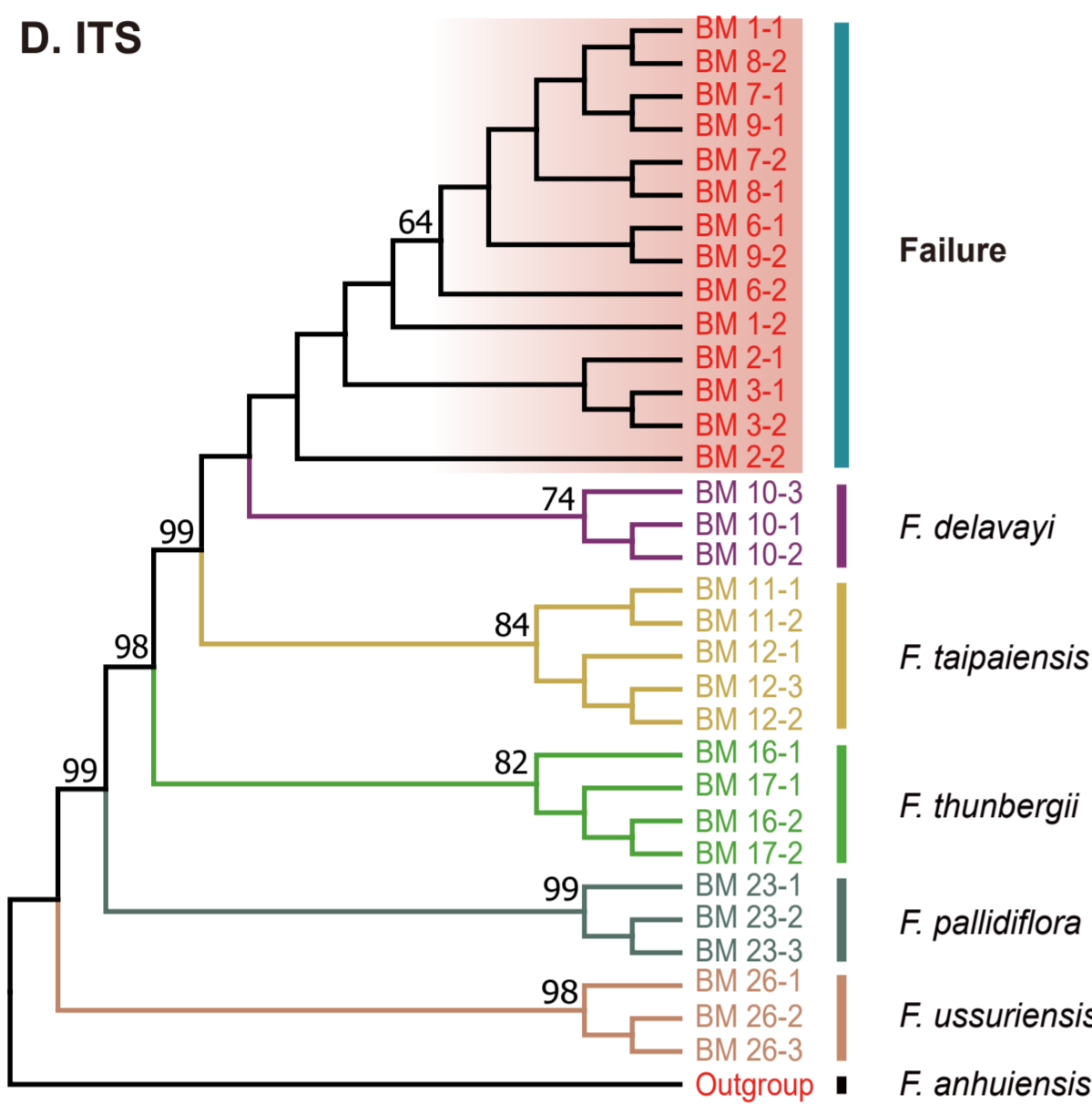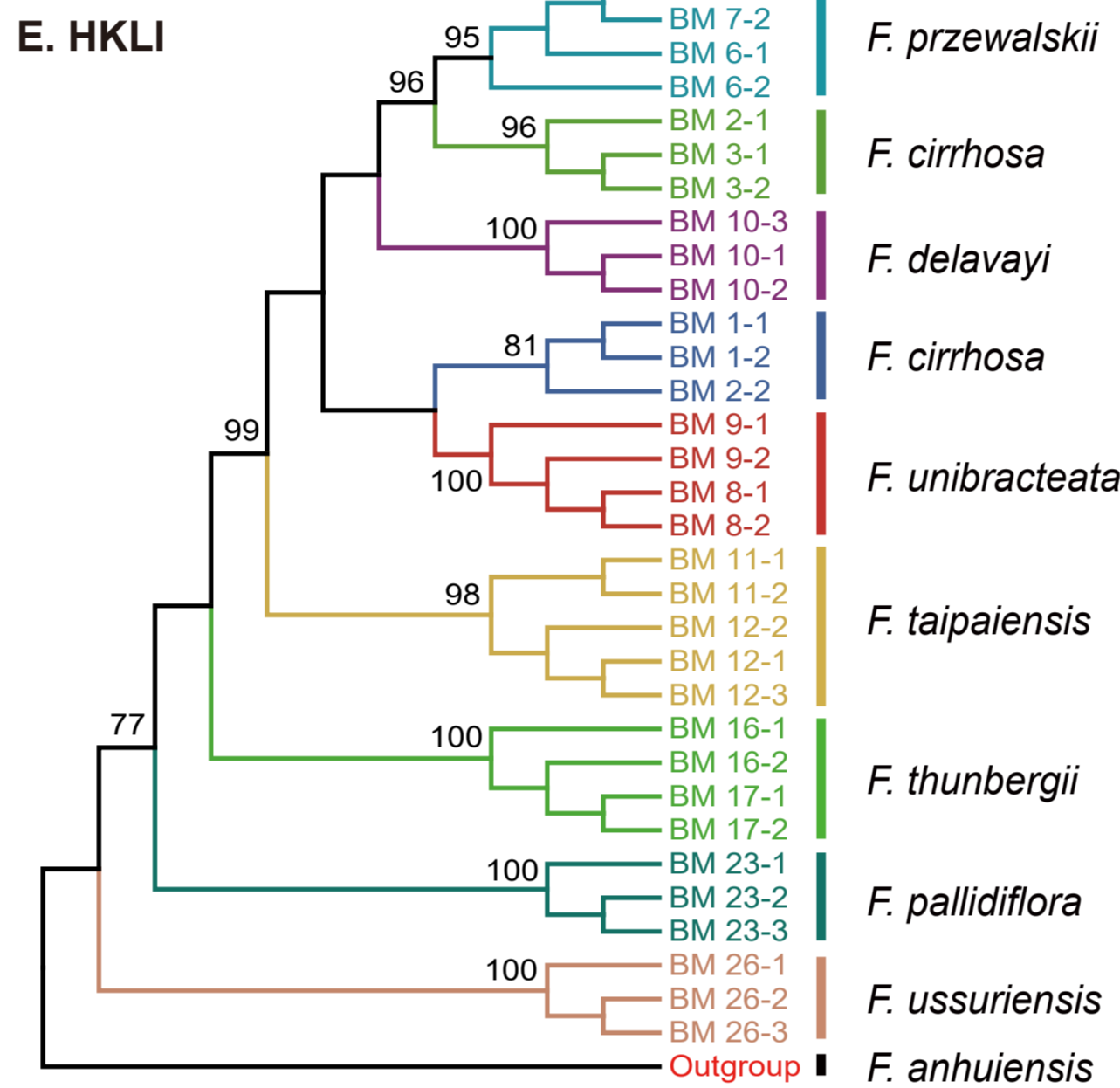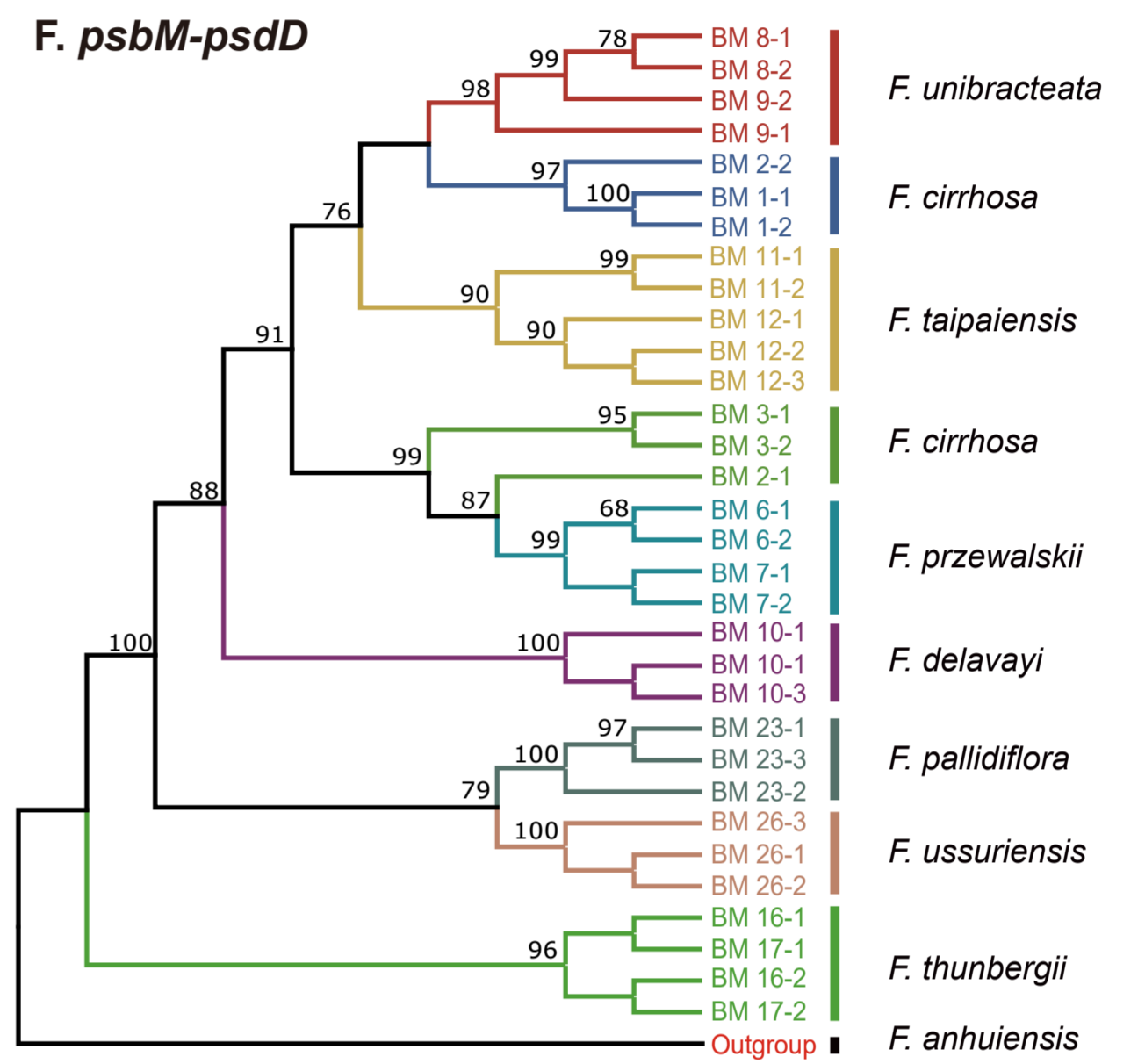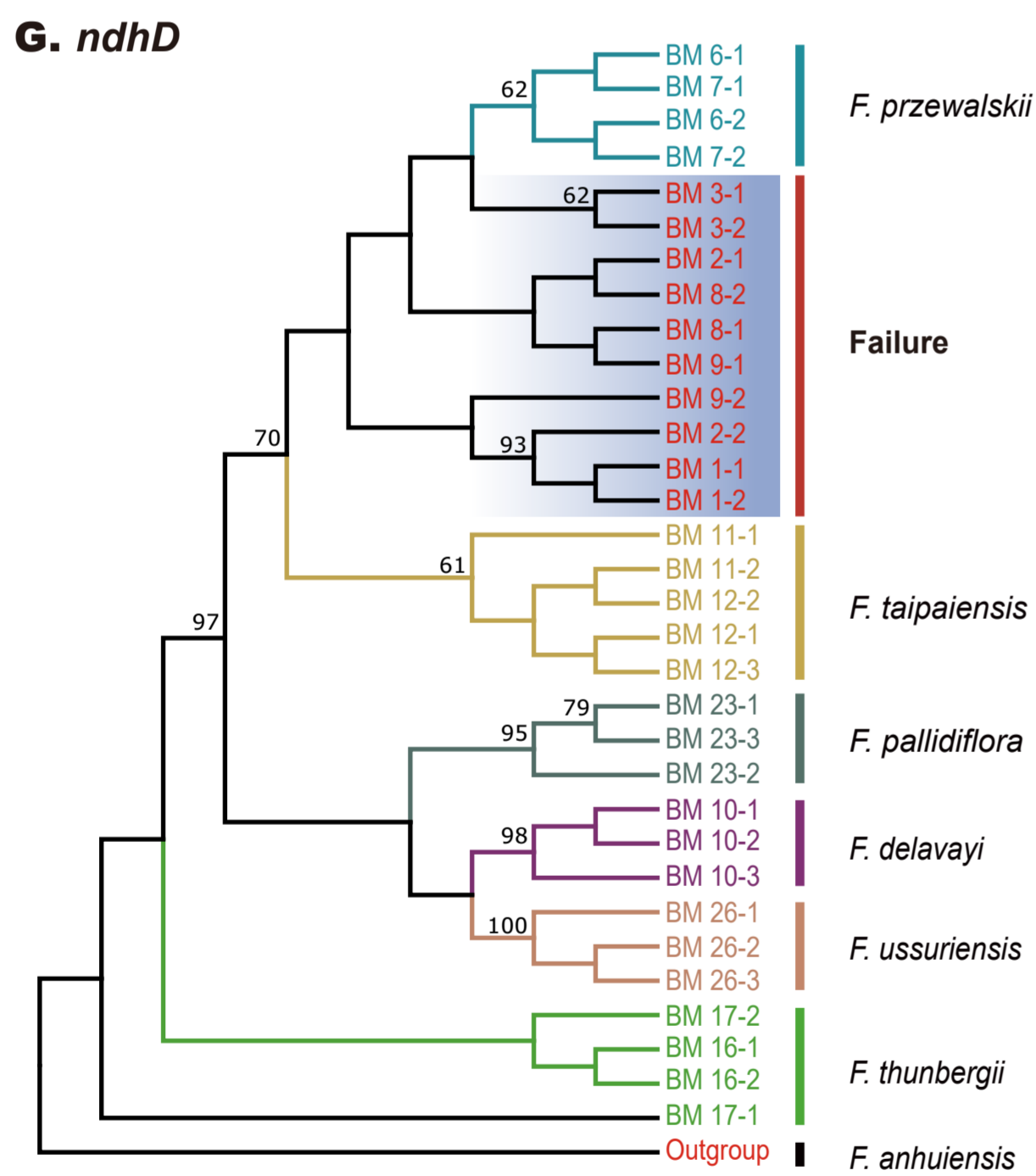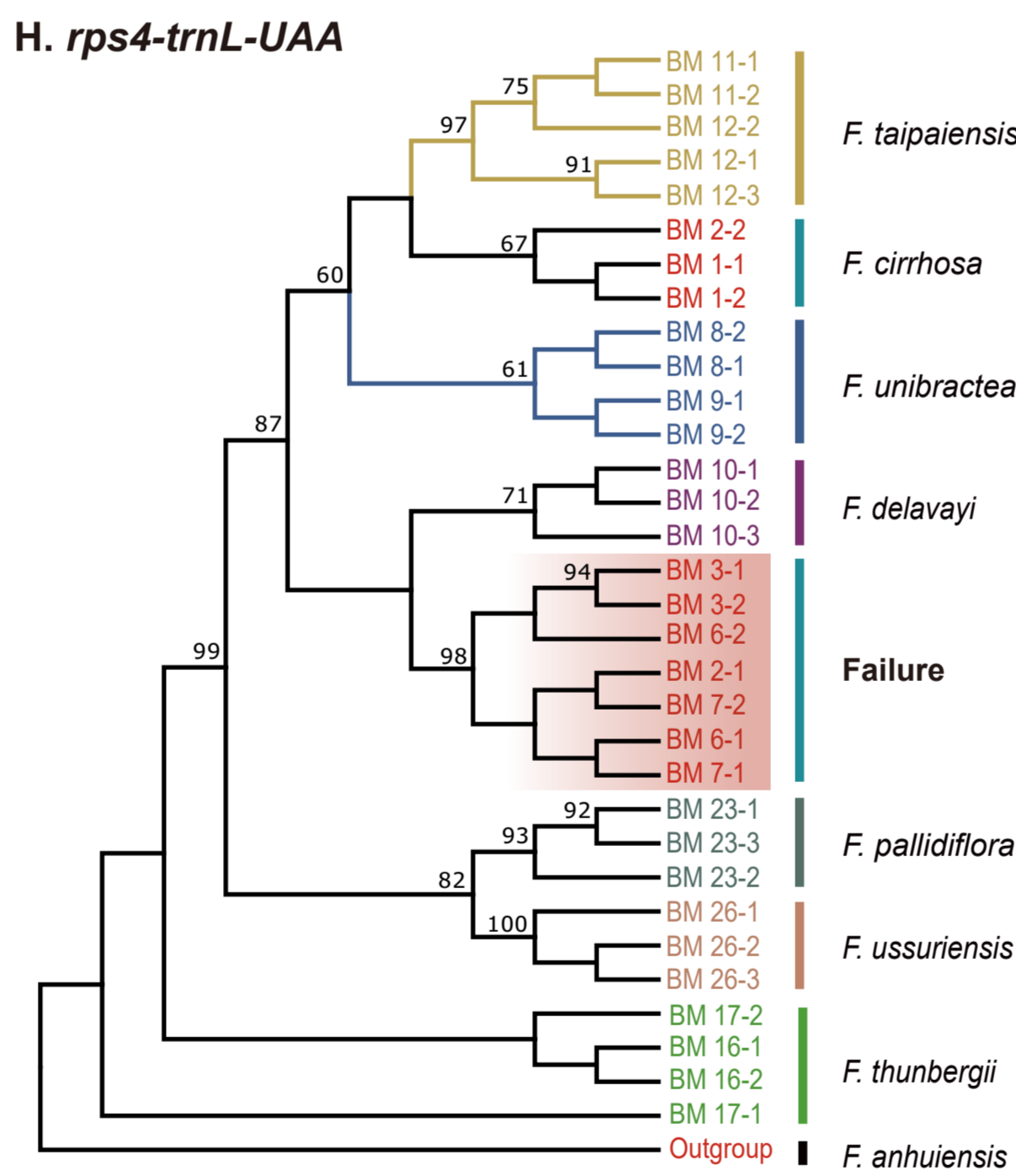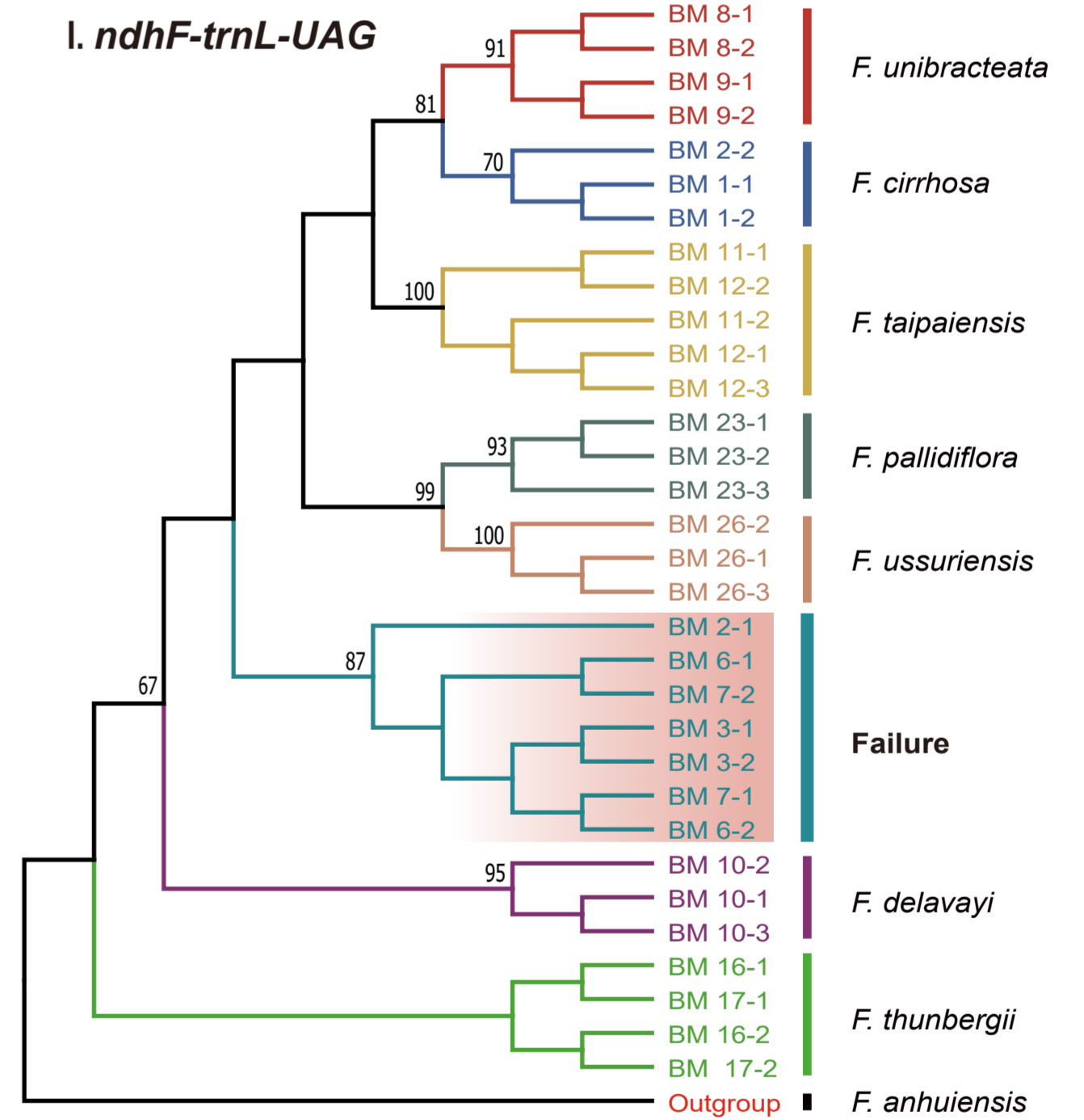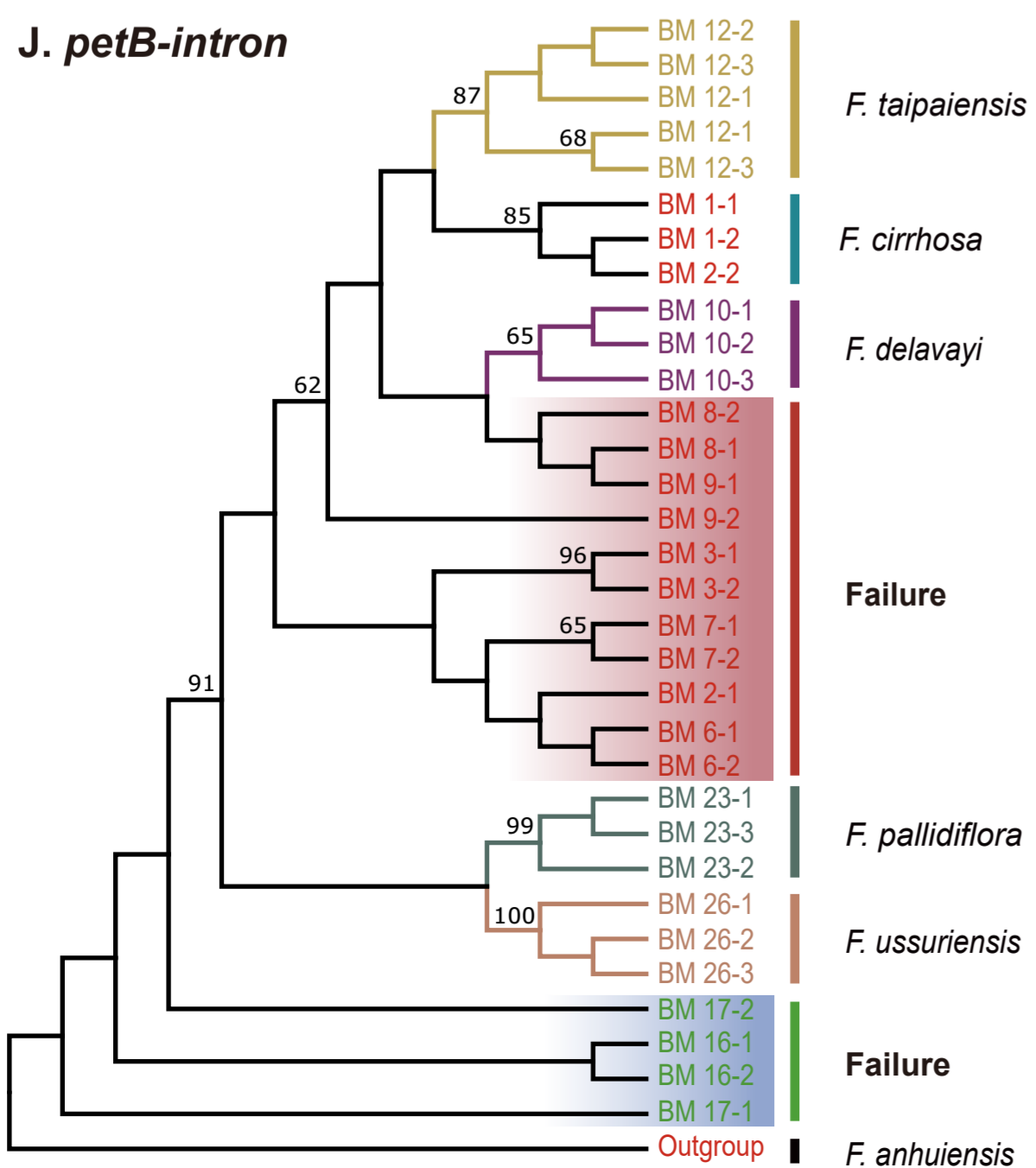

Supplement: S3 Fig — (H: trnH-psbA; K: matK; L: rbcL; I: ITS) (PDF) (PDF) [file pone.0229181.s003.pdf]
