# Supplementary material for: Comparison of the abilities of universal, super, and specific DNA barcodes to discriminate among the original species of Fritillariae cirrhosae bulbus and its adulterants
Source: PLoS One. 2020 Feb 13;15(2):e0229181. doi: 10.1371/journal.pone.0229181 (PMC7018091; doi:10.1371/journal.pone.0229181)
Supplement: S1 Table — (DOCX) [file pone.0229181.s004.docx]

**Table S1 | Collecting information of *Fritillaria* species in this study**

| Species | Number | Locality | Latitude/Longitude | Altitude(m) | Voucher specimen |
| --- | --- | --- | --- | --- | --- |
| *F. cirrhosa* | BM 1-1 | Lijiang, Yunnan, China | N27°03.57′/E100°14.13′ | 3142 | ZDQ15019 |
|  | BM 1-2 |  |  |  |  |
|  | BM 2-1 | Shangri-La, Yunnan, China | N28°08.100′/E99°52.880′ | 4212 | ZDQ13053 |
|  | BM 2-2 |  |  |  |  |
|  | BM 3-1 | Basu, Xizang, China | N29°38.636′/E96°42.856′ | 4480 | ZDQ14027 |
|  | BM 3-2 |  |  |  |  |
| *F. przewalskii* | BM 6-1 | Ganzi, Sichuan, China | N31°33.164′/E100°00.926′ | 3682 | ZDQ13018 |
|  | BM 6-2 |  |  |  |  |
|  | BM 7-1 | Ganzi, Sichuan, China | N31°45.895′/E100°45.653′ | 4047 | ZDQ13029 |
|  | BM 7-2 |  |  |  |  |
| *F. unibracteata* | BM 8-1 | Hongyuan, Sichuan, China | N32°10.532′/E102°30.686′ | 3621 | ZDQ13030 |
|  | BM 8-2 |  |  |  |  |
|  | BM 9-1 | Songpan, Sichuan, China | N32°53.419′/E103°30.390′ | 3199 | ZDQ13032 |
|  | BM 9-2 |  |  |  |  |
| *F. delavayi* | BM 10-1 | Lijiang, Yunnan, China | N27°03.52′/E100°11.81′ | 4071 | ZDQ17003 |
|  | BM 10-2 |  |  |  |  |
|  | BM 10-3 |  |  |  |  |
| *F. taipaiensis* | BM 11-1 | Wuxi, Chongqing, China | N31°33.865′/E109°06.490′ | 2230 | ZDQ19022 |
|  | BM 11-2 |  |  |  |  |
|  | BM 12-1 | Foping, Shanxi, China | N33°36.7′/E107°48.418′ | 1470 | ZDQ15017 |
|  | BM 12-2 |  |  |  |  |
|  | BM 12-3 |  |  |  |  |
| *F. thunbergii* | BM 16-1 | Dongyang, Zhejiang, China | N29°01.183′/E120°20.833′ | 230 | ZDQ15009 |
|  | BM 16-2 |  |  |  |  |
|  | BM 17-1 | Nantong, Jiangsu, China | N31°55.77′/E121°00.23′ | 5 | ZDQ16017 |
|  | BM 17-2 |  |  |  |  |
| *F. pallidiflora* | BM 23-1 | Gongliu, Xinjiang, China | N43°12.924′/E82°36.285′ | 1178 | ZDQ16011 |
|  | BM 23-2 |  |  |  |  |
|  | BM 23-3 |  |  |  |  |
| *F. ussuriensis* | BM 26-1 | Hengyuan, Liaoning, China | N41°20.466′/E125°17.069′ | 275 | ZDQ16015 |
|  | BM 26-2 |  |  |  |  |
|  | BM 26-3 |  |  |  |  |
| Outgroup |  |  |  |  |  |
| *F. anhuiensis* | BM 20-2 | Guangde, Anhui, China | N30°56.383′/E119°14.817′ | 16 | ZDQ15011 |
